# Multi-dimensional resilience: A quantitative exploration of disease outcomes and economic, political, and social resilience to the COVID-19 pandemic in six countries

**DOI:** 10.1371/journal.pone.0279894

**Published:** 2023-01-05

**Authors:** Lauren J. Beesley, Paolo Patelli, Kimberly Kaufeld, Jon Schwenk, Kaitlyn M. Martinez, Travis Pitts, Martha Barnard, Ben McMahon, Sara Y. Del Valle

**Affiliations:** 1 Information Systems & Modeling, Los Alamos National Laboratory, Los Alamos, New Mexico, United States of America; 2 Statistical Sciences, Los Alamos National Laboratory, Los Alamos, New Mexico, United States of America; 3 Earth Systems & Observations, Los Alamos National Laboratory, Los Alamos, New Mexico, United States of America; 4 Intelligence & Systems Analysis, Los Alamos National Laboratory,Los Alamos, New Mexico, United States of America; 5 Theoretical Biology & Biophysics, Los Alamos National Laboratory, Los Alamos, New Mexico, United States of America; Sri Lanka Institute of Information Technology, SRI LANKA

## Abstract

The COVID-19 pandemic has highlighted a need for better understanding of countries’ vulnerability and resilience to not only pandemics but also disasters, climate change, and other systemic shocks. A comprehensive characterization of vulnerability can inform efforts to improve infrastructure and guide disaster response in the future. In this paper, we propose a data-driven framework for studying countries’ vulnerability and resilience to incident disasters across multiple dimensions of society. To illustrate this methodology, we leverage the rich data landscape surrounding the COVID-19 pandemic to characterize *observed* resilience for several countries (USA, Brazil, India, Sweden, New Zealand, and Israel) as measured by pandemic impacts across a variety of social, economic, and political domains. We also assess how observed responses and outcomes (i.e., resilience) of the COVID-19 pandemic are associated with pre-pandemic characteristics or vulnerabilities, including (1) prior risk for adverse pandemic outcomes due to population density and age and (2) the systems in place prior to the pandemic that may impact the ability to respond to the crisis, including health infrastructure and economic capacity. Our work demonstrates the importance of viewing vulnerability and resilience in a multi-dimensional way, where a country’s resources and outcomes related to vulnerability and resilience can differ dramatically across economic, political, and social domains. This work also highlights key gaps in our current understanding about vulnerability and resilience and a need for data-driven, context-specific assessments of disaster vulnerability in the future.

## 1 Introduction

The ongoing novel coronavirus (also referred to as COVID-19) pandemic and associated economic crisis have impacted every corner of the earth, pushing millions of people into poverty, disrupting governments, and leading to socioeconomic instability around the globe. The pervasive and consequential impacts of the pandemic present us with a unique opportunity to quantify and analyze societal responses to the pandemic and evaluate resilience by comparing pandemic outcomes between communities exposed to the same threat. Understanding and quantifying nationwide resilience can help us determine how prepared or vulnerable countries are to different threats and potentially inform preemptive intervention.

The concepts of vulnerability and resilience have been studied extensively in the contexts of climate change [1, 2], disaster preparedness [3], and sustainability [4], but limited studies have focused on pandemic resilience at the national or sub-national level. The definitions of these abstract concepts have also varied substantially. For example, some analysts have defined vulnerability/resilience as a single concept representing a composite of (1) a country’s essentially *unchangeable* prior sensitivity to adverse outcomes of a shock/disaster (e.g., due to physical location, population size, climate) and (2) a country’s capacity to adapt or respond in terms of financial resources and infrastructure [1, 5, 6]. Others define vulnerability and resilience separately, where resilience represents the ability of a system to absorb shocks related to disasters, while vulnerability often describes intrinsic quasi-permanent characteristics of a country (e.g., the age of the population) that may impact sensitivity to poor outcomes of a shock or the likelihood of experiencing a shock [2, 4]. The United Nations International Strategy for Disaster Reduction [7] defines resilience as “the ability of a system, community or society exposed to hazards to resist, absorb, accommodate, adapt to, transform and recover from the effects of a hazard in a timely and efficient manner, including through the preservation and restoration of its essential basic structures and functions through risk management.” Additionally, adaptive capacity, or the resources and infrastructure available to respond to a shock, is often incorporated into the definitions of either vulnerability or resilience. In this work, we define vulnerability and resilience as distinct concepts, where we define **vulnerability** as a composite of (1) a country’s prior risk/sensitivity to poor pandemic outcomes and (2) a country’s capability of response to the pandemic. **Resilience** is defined in terms of countries’ *observed* (rather than *hypothetical*) response to the COVID-19 pandemic across a variety of social, economic, and political domains, and our primary focus will be characterizing resilience and exploring associations with pre-pandemic vulnerabilities.

In general, the majority of the literature focuses on *qualitative* assessments of vulnerability/resilience. For example, Moore et al. (2016) [6] developed an infectious disease vulnerability index (IDVI), a leading index of multi-domain resilience for infectious diseases. This index was developed by synthesizing *expert opinion* on 53 countries’ vulnerability/resilience. Their evaluation aggregates country characteristics and qualitative subject matter expertise to describe *hypothetical* resilience to a pandemic. The INFORM Epidemic Risk Index (ERI) similarly leverages expert opinion to construct scores characterizing countries’ resilience [8]. This and other related indices are very useful for consolidating expert knowledge into concise data metrics. A comparatively small amount of work has focused on *quantitative* assessments of (usually economy-related and/or spatially localized) vulnerability and resilience by measuring these quantities using data collected before, during, and after an observed disaster [9–11]. These analyses can provide valuable insight into *observed* rather than *hypothetical* disaster response, which can help reveal gaps in our current understanding about vulnerability and resilience.

In this paper, we leverage the rich data landscape surrounding the COVID-19 pandemic to characterize *observed* resilience for several countries as measured by pandemic impacts across a variety of social, economic, and political domains in USA, Brazil, India, Sweden, New Zealand, and Israel. We also study how observed responses and outcomes of the COVID-19 pandemic are associated with pre-pandemic characteristics or vulnerabilities, including (1) prior risk for adverse pandemic outcomes due to population density and age and (2) the systems in place prior to the pandemic that may impact the ability to respond to the crisis, including health infrastructure and economic capacity. Our work demonstrates the *importance of viewing vulnerability/resilience in a multi-dimensional way*, where a country’s resources and outcomes related to economic, political, and social vulnerability and resilience can differ dramatically. This paper also highlights key gaps in our current understanding about vulnerability and resilience and a *need for data-driven, context-specific assessments* of disaster vulnerability in the future.

In **Section 2**, we describe the variety of data sources used to construct data proxies for pre-pandemic vulnerability and observed pandemic resilience. We explain our conceptual vulnerability-resilience framework and statistical analysis in **Section 3**. Results are presented in **Section 4**, and we provide a discussion and conclusion in **Section 5**.

## 2 Data sources and regions of interest

We collected six types of data including clinical, political and social unrest, mobility, governmental policy, economic, and demographic data across various spatial and temporal resolutions for the six countries of interest: USA, India, Brazil, New Zealand, Israel, and Sweden. These six countries were chosen due to their differing geographic regions, pandemic responses and outcomes, and sociodemographic characteristics. Combined, data from these six countries are used to develop and demonstrate the proposed generalizable approach to quantifying resilience in a holistic, multi-dimensional way.

**Fig 1:**
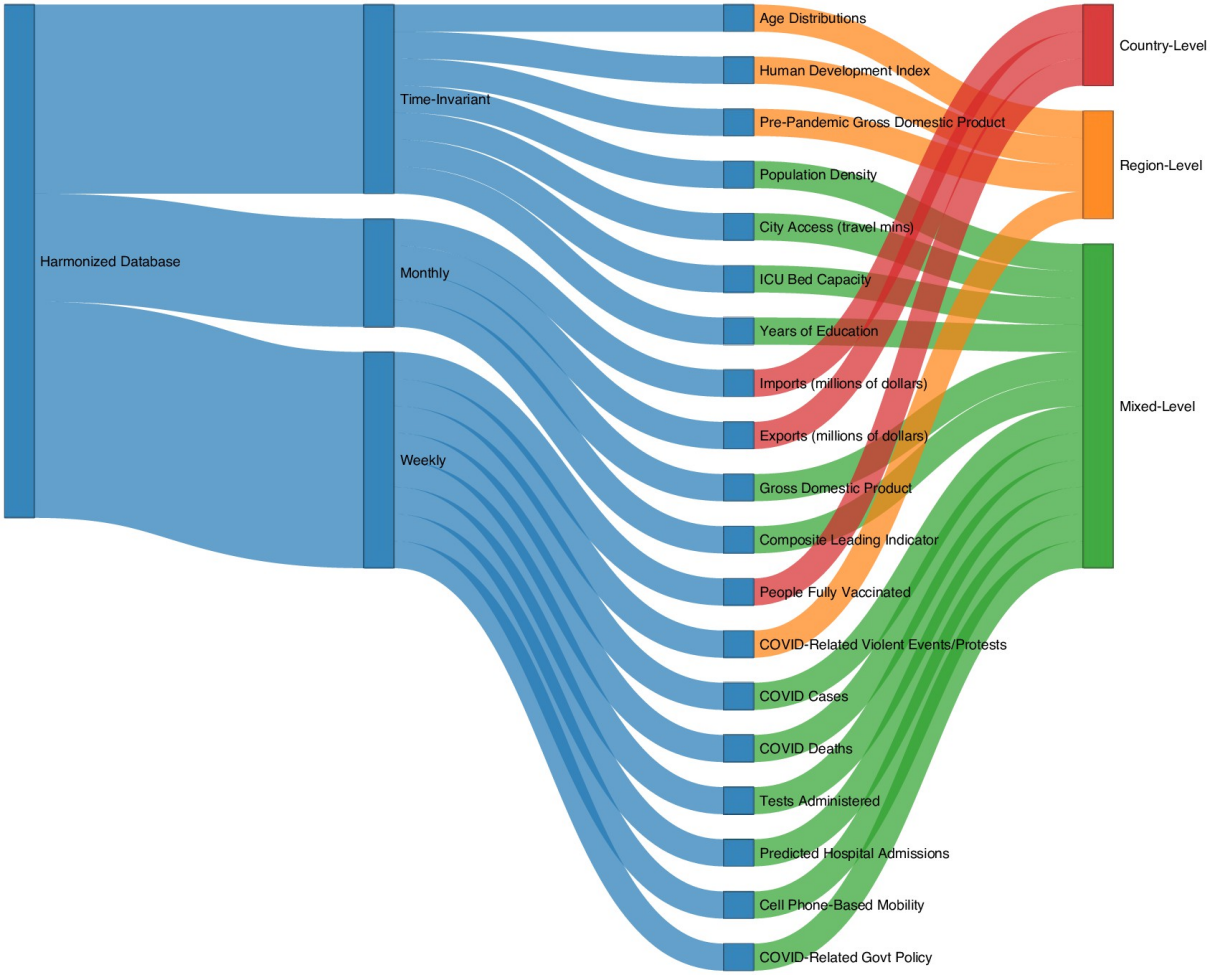
Overview of data types, spatial, and temporal resolutions^1^. ^1^ Spatial resolutions correspond to available data; data may be entirely unavailable for some countries. Additional details can be found in **S1 Table A.2** and **S1 Section A** in S1 File. Mixed-level spatial resolution indicates that administrative level 0 data is available for some countries and administrative level 1 data is available for others.

To facilitate our data analyses, these data were harmonized to the weekly temporal resolution (for time-varying variables) and to state/region-level spatial resolution when available. Spatial resolutions were defined by administrative levels, where administrative level 0 corresponds to countries and administrative level 1 corresponds to regions as defined by the United Nations Second Administrative Level Boundaries (2021) [12], where administrative level 1 regions are nested within administrative level 0 countries. Fig 1 summarizes the types of data available and their spatial and temporal resolutions.

### 2.1 Clinical data and outcomes

Daily confirmed COVID case and COVID-related death data were obtained from the John’s Hopkins Center for Systems Science and Engineering [13]. These data were available at administrative level 1 from February or March 2020 through June 2021 for the US, Sweden, India, and Brazil and were available at administrative level 0 for Israel and New Zealand.

Data on COVID tests administered were made publicly available by the Institute for Health Metrics and Evaluation (IHME) at The University of Washington [14]. In addition, IHME model-predicted hospital admissions were used in this analysis to represent true hospital admissions. Data were available between February 2020 and June/July 2021. Administrative level 1 data were available for Brazil, the US, and India, and administrative level 0 data were available for other countries. Pre-pandemic ICU bed capacity was also provided by IHME at administrative level 1 for US and Brazil and at administrative level 0 for all other countries. Administrative level 0 COVID vaccination rates between January and June 2021 were obtained from Our World in Data [15].

### 2.2 Political and social unrest

Political and social unrest data were obtained from the Armed Conflict Location & Event Data Project (ACLED), a non-profit organization that collects data on protests and political violence from traditional media outlets, non-governmental organizations, social media, and local partner organizations [16]. ACLED data were coded by date, actor, location, and event type. Event types include protests, four types of violent events, and strategic developments or “contextually important data regarding the activities of violent groups.” A field labeled “notes” was also included that contained event descriptions. COVID-19-related events were identified as those with the following search terms/fragments present in the notes: “covid”, “lockdown”, “mask”, “vaccin”, and “quarantin.” A composite score was constructed for each week and administrative 1 level as the total number of recorded ACLED events (representing protests, violent events, and strategic developments). Data were collected at administrative level 1 for all six countries of interest between November 2019 and March 2021 with the exception of New Zealand, for which data were not available from ACLED.

### 2.3 Mobility

A composite score representing cell phone-based mobility was provided by the IHME [14]. This mobility composite was based on anonymous cell phone data from Descartes and Safegraph for the US and on Google, Facebook, and Apple data elsewhere. The mobility composite score for a given location and week represents the percent change in mobility relative to a single baseline value for that location, defined in January and early February, 2020. Mobility data were available at administrative level 1 between mid-February 2020 and June 2021 for the US, Brazil, and India and at administrative level 0 for Israel, New Zealand, and Sweden.

### 2.4 Governmental policy

Government response data were collected from the Oxford Covid-19 Government Response Tracker (OxCGRT), a project by Oxford University’s Blavatnik School of Government that collects publicly available data on the measures governments have taken in response to COVID-19 [17]. OxCGRT records policy responses on an ordinal scale to reflect the extent of government action for each category of response (e.g., social distancing restrictions, travel policies, mask mandates). Scores are aggregated into four different policy indices: 1) overall government response, 2) containment and health, 3) stringency, and 4) economic support. These indices are simple averages of the response categories and measure how many of the relevant policy responses a government has implemented and to what degree. In this analysis, we considered only the overall governmental response indicator, which is measured on a 0 (least strict) to 100 (most strict) scale. This index averages all policies tracked by OxCGRT, namely closure, health, and economic policies. Data were collected between January 2020 and May 2021 at administrative level 1 for Brazil and the United States and at administrative level 0 for India, Israel, New Zealand, and Sweden.

### 2.5 Economic data

Macro and microeconomic data and financial time-series were collected from multiple sources. The Organisation for Economic Co-operation and Development (OECD) provided global economic data (e.g., GDP, unemployment), key economic indicators (e.g., industrial production, retail volumes, earnings, consumer prices), and the Composite Leading Indicators (CLI) [18]. OECD also provided monetary variables including broad money and various interest rates. OECD administrative level 0 data were not available for New Zealand, and metrics were reported at the monthly level.

Administrative level 1 data for GDP in the US were obtained from The Bureau of Economic Analysis (BEA) through the Federal Reserve of St. Louis [19]. Import-export data aggregated at the administrative level 0 and economic sector levels are produced and published by the World Trade Organization (WTO) at the monthly time resolution [20]. Exports data were not available for New Zealand. We obtained administrative level 1 data on unemployment in the US from The Bureau of Labor Statistics at the monthly time resolution [21]. We obtained daily time series for the following market indexes from Google Finance: S&P500 for the USA, OMX for Sweden, BVSP for Brazil, BSESN for India, TA125 for Israel, and NZ50 for New Zealand. The Global Multiregional Input-Output Framework database [22, 23] provided the harmonized economic input-output tables at the administrative 1 level.

### 2.6 Demographics and other data sources

Pre-pandemic demographic information included estimates of human population in 2010 by age and sex were derived from the Gridded Population of the World [24], which provides these data globally at a spatial resolution of 30 arc-seconds (roughly 1 km at the equator). Pre-pandemic gross domestic product (GDP) and human development indices (HDI) were derived from global, gridded rasters created by Kummu et al. (2018) [25]. GDP was provided at a resolution of 30 arc-seconds, while HDI was available at a 5 arc-min spatial resolution (roughly 9 km at the equator).

All datasets provided as geospatial rasters were aggregated to the appropriate administrative boundary regions using Google Earth Engine [26] and administrative boundary shapefiles obtained from GADM [27]. Typically, simple averaging was used to aggregate gridded data to an administrative polygon with nearest neighbor resampling. In some cases, aggregation was performed via sums rather than averages (e.g., population counts).

Data on average years of schooling were obtained from [28], where administrative level 1 metrics were available for the USA, Brazil, New Zealand, and India, while administrative level 0 metrics were available for Israel and Sweden.

## 3 Methods

### 3.1 Conceptual model of vulnerability and resilience

**Fig 2:**
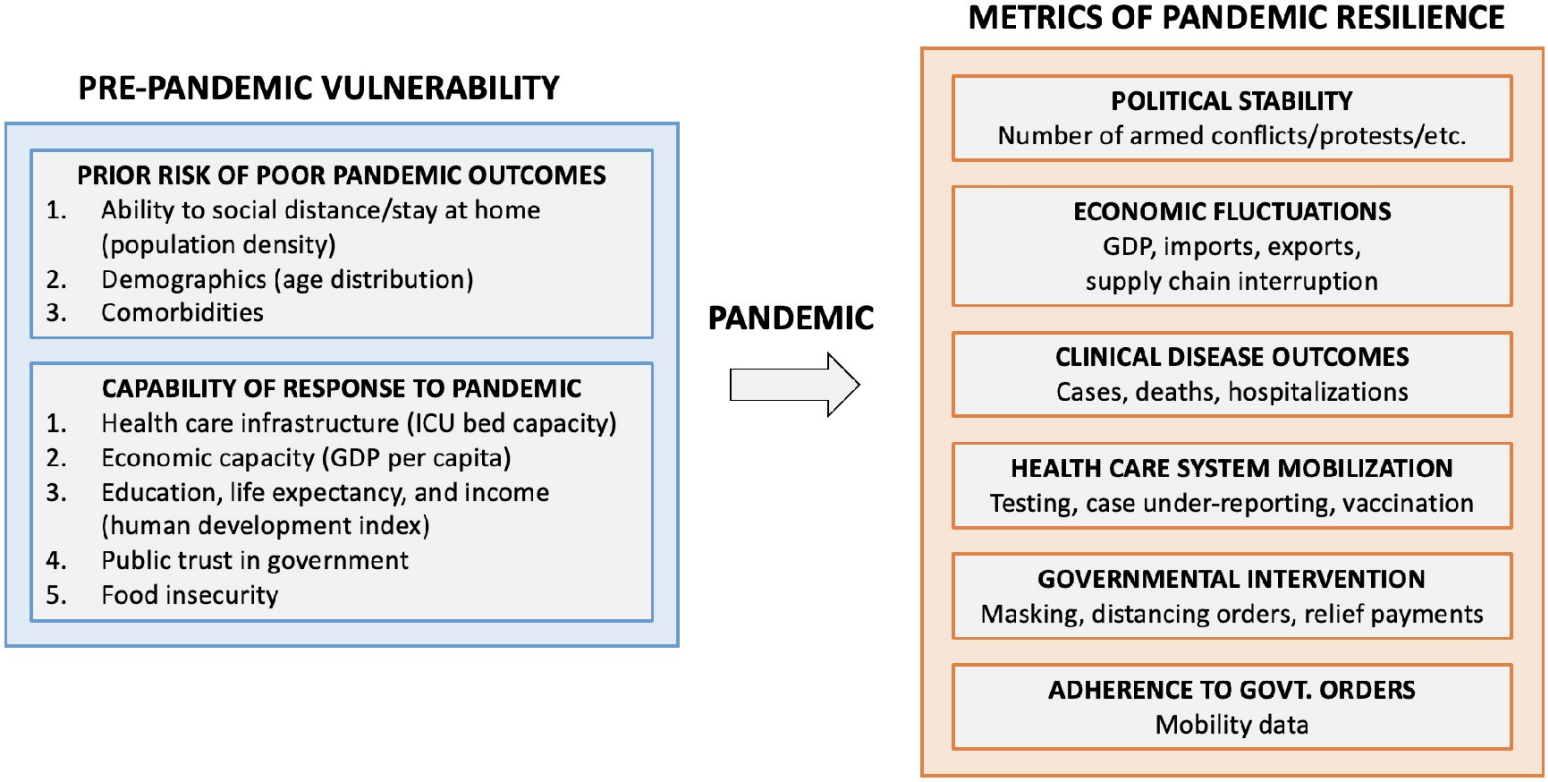
Conceptual diagram of vulnerability and resilience^1^. ^1^ Our conceptual model of pandemic vulnerability (left) and resilience (right). Data we used to measure vulnerability variables are in parenthesis; not all of our conceptual vulnerability domains have corresponding proxy data.

In this study, we propose a quantitative framework for exploring vulnerability and resilience using data from the COVID-19 pandemic. Much like Moore et al. (2016) [6], we define **vulnerability** as a composite of (1) a country’s prior risk/sensitivity to poor pandemic outcomes and (2) a country’s capability of response to the pandemic. *Prior risk* includes the ability of a population to physically social distance and the baseline health and age of the population, both of which are expected to be related to disease outcomes among infected individuals. A country’s *ability to respond* is related to its health care infrastructure and its economic capacity. Crucially, the ability of a country to effectively respond to a pandemic may also be a function of the relationship between governmental policy and public adherence/response, which may further depend on education levels and public trust. In our conceptual model (Fig 2), these pre-pandemic characteristics in aggregate define a country’s vulnerability to the COVID-19 pandemic. In practice, data proxies are used to represent different dimensions of pre-pandemic vulnerability.

Moore et al. (2016) [6] defines resilience as the opposite of vulnerability, where the two terms are used to describe the same concept that implicitly relates to *hypothetical* impacts of crises on a country. In contrast, we define **resilience** in terms of the *observed* response of a country to the COVID-19 pandemic across a variety of social, political, and economic dimensions as follows:

1. *Economic fluctuations:* Fluctuations in GDP, supply chain interruption, imports, and exports relative to pre-pandemic levels measure economic resilience, where smaller fluctuations and faster recovery represent greater resilience to the market shock created by the pandemic.
2. *Political stability:* Levels of political unrest related to COVID-19 reflect the interplay between governmental response and public perceptions. We use the number of pandemic-related civil unrest events (e.g., protests and riots) as a proxy for the resilience of the political system in terms of crisis response appropriateness, indirectly measured by the amount of public backlash.
3. *Health care system mobilization:* Pandemic resilience is also defined by the ability of a country to mobilize its health care system to respond effectively to the pandemic. The mobilization/resilience of the health care system is characterized in terms of COVID testing rates, vaccination uptake, and estimated under-reporting of COVID-19 cases.
4. *Governmental intervention:* We define resilient governmental policies as those that “appropriately” react to the pandemic in terms of lockdowns, mask mandates, etc. “Appropriateness” is of course a subjective function of the current disease burden and a country’s prioritization of various competing factors. Therefore, we assess appropriateness in terms of the strictness of governmental policy early in the pandemic and responsiveness as case rates rose throughout the pandemic.
5. *Population adherence to government orders:* While government policy may be well-calibrated and responsive to current infection rates, it will only be effective at controlling an ongoing pandemic if the population complies. We thus define another dimension of resilience measuring the interplay between governmental orders and population compliance using cell phone-derived mobility data. Rising policy strictness paired with little reduction in mobility indicates a lack of population adherence.
6. *Clinical disease outcomes:* The aggregate response of a country in terms of the disease burden is measured in terms of case, hospitalization, and death incidence (all normalized by population size), where lower population-adjusted incidence represents more resilient overall country response to the pandemic in terms of disease burden. Confirmed clinical outcomes are evaluated in conjunction with estimated under-reporting rates, where low confirmed case counts may be a result of low disease burden or poor case catchment.

A key difference between our approach and existing work is that we consider data measured before and during a shock to measure the *observed* rather than *hypothetical* country resilience. While some works such as Brada et al. (2021) [9] have evaluated country resilience to the COVID-19 pandemic in terms of economic outcomes, we assess data metrics of resilience across a variety of conceptual domains in an attempt to capture resilience in a more holistic way.

### 3.2 Statistical methods

#### Estimating COVID case under-reporting, a proxy for health care system resilience

We estimated the amount of case under-reporting for each country over time by leveraging observed COVID-19 case and death rates using a modification of the method in Lau et al. (2020) [29]. Under-reporting factors were estimated as the (smoothed in time) ratio between observed case fatality rates and age-adjusted “true” infection fatality rates reported by O’Driscoll et al. (2021) [30]. A sensitivity analysis accounting for vaccination status was also explored. Additional details are provided in **S1 Section B in** S1 File. This estimation does not account for differences in comorbidities across countries after adjusting for age. The primary analysis also does not account for potential changes in the infection-fatality ratio in vaccinated individuals, which may result in under-estimates of the under-reporting factors. Future studies should consider modified target infection-fatality ratios due vaccination in more detail.

#### Characterizing resilience metrics and their unadjusted associations

Using the harmonized data, we characterize resilience metrics within each country based on visual assessment of trends, and we attempt to contextualize temporal changes in these metrics based on country characteristics and policy decisions. We also calculate cross correlations between contemporaneous resilience metrics within countries to quantify how strongly different resilience metrics are associated. To assess how these relationships changed over time, we calculated the time series cross correlations stratified by calendar quarter.

Correlations of resilience metrics in the same week will not adequately capture time-lagged associations such as the relationship between current COVID cases and future COVID-related deaths, and it may be sensitive to induced correlation due to general trends over time. To more rigorously assess the relationships between resilience metrics, we fit a linear model for current values for a given resilience metric A (e.g., log-cases per 1000), adjusting for the previous 3 weeks’ values for metric A and also the previous 3 weeks’ values of *another* resilience metric B (e.g., log-deaths per 1000). For cases/deaths/hospitalizations/testing outcomes and COVID under-reporting rate estimates, these resilience metrics were log-transformed prior to model fitting. We then performed a Wald statistical test (called a “Granger test”) for whether past values of metric B (e.g., deaths) are associated with current reported values of metric A, adjusting for past values of metric A. Given the large potential for additional confounding (i.e., other variables related to both variable A and variable B), we do not interpret the results of these tests in terms of causality. For each country, these test results were visualized using network diagrams, where an arrow going from metric *B* to metric *A* represents a statistically significant Granger association at *α* = 0.01.

#### Mixed modeling of resilience

The correlation and Granger test analyses evaluate associations between resilience metrics *without adjustment for pre-pandemic vulnerability. To evaluate the adjusted relationship between time-constant and time-varying administrative level 1 (regional) vulnerability characteristics and the time-varying resilience metrics, we performed a mixed modeling analysis using two different models specifications:*(1) For each resilience outcome, we first fit a linear mixed model to the time and space-varying resilience metrics for each country. This mixed model included a random intercept for region and accounted for temporal correlation within each region through an auto-regressive moving average model for the errors, and (2) we then fit a Bayesian spatio-temporal model using the R package *CARBayesST* [31], where the spatial relationships between neighboring regions were accounted for in terms of a spatial process adjacency matrix indicating bordering regions and where temporal correlations were accounted for using a simple conditional autoregressive model. A autoregressive error structure was used in both models to account for autocorrelation in resilience metrics over time. Differences between these model specifications provide insight into the impact of accounting for spatial relationships between neighboring regions on vulnerability-resilience and resilience-resilience associations. We present parameter estimates from these mixed models along with corresponding 95% confidence or credible intervals.

### Input-output modeling to estimate pandemic impact on economic sectors

We then conducted a more extensive characterization of economic resilience within each country, leveraging existing knowledge about supply and labor structures within each country. We modeled the impact of the pandemic on various economic sectors in each country based on input-output relations between industries, which are commonly used to study the effects of a shock (i.e., the pandemic) on the economy [32]. These models captured both the direct effect of the shocks as well as secondary effects expressed through interconnected industries. For example, an increase in final demand for an industry’s output will spur output production in other sectors, a dynamic phenomenon known as the “output multiplier effect” [33].

We adopt a pure input-output analysis [33], which allows for *comparisons across multiple countries*. For this analysis, we used the *Global Multiregional Input-Output (GMRIO)* input-output tables from Timmer et al. (2015) [22], which incorporate connectivity between countries through import-export relations. Using the GMRIO input-output tables at administrative level 0, we calculated the technical coefficients matrix for each country and the Leontief inverse matrix (i.e., the industries’ impact multipliers). See **S1 Section F.1 in** S1 File for details. We then performed the following analysis:

1. Using the multiplier matrix, we simulated the direct effect of the pandemic in terms of a workforce reduction on the various economic sectors. The probability of a worker getting infected was allowed to differ across industries. For example, a worker in the health care industry has a higher probability of getting infected than a worker in the information technology sector. The methodology and data sources we used to measure the relative change in infection rate across industries are described in **S1 Section F.2 in** S1 File. Data regarding COVID-19 incidence in different industrial sectors were not available for all countries; for this reason, we assumed that the infection rate per industry sector was the same for all the countries that were part of the analysis. For simplicity, we also assumed that the workers’ age distribution did not differ across industrial sectors.
2. Using the daily number of new COVID cases for each country and assuming that an infected worker was unable to work for three weeks on average, we calculated the daily number of idle workers per industry between February, 2021 and July, 2021.
3. The number of idle workers was then transformed, using the labor productivity data of each industry, into a contraction of the economic output in each sector. These contractions were finally propagated through the IO model to calculate financial losses (dollars) to the national economy attributable to that sector.

For more details about the input-output modeling approach and our analysis, see **S1 Sections F.1 and F.3 in** S1 File.

## 4 Results

In this section, we characterize countries’ resilience to the COVID-19 pandemic across multiple dimensions (e.g., political, economic) and examine the interplay between different aspects of resilience. Then, we explore how observed pandemic resilience is associated with pre-pandemic vulnerability to poor COVID-19 outcomes and societal infrastructure/capability of response. Finally, an input-output analysis allows us to quantify the relative monetary impact of the COVID-19 pandemic on different economic sectors within and between countries.

### 4.1 Visual characterization of resilience at the country level

**Fig 3:**
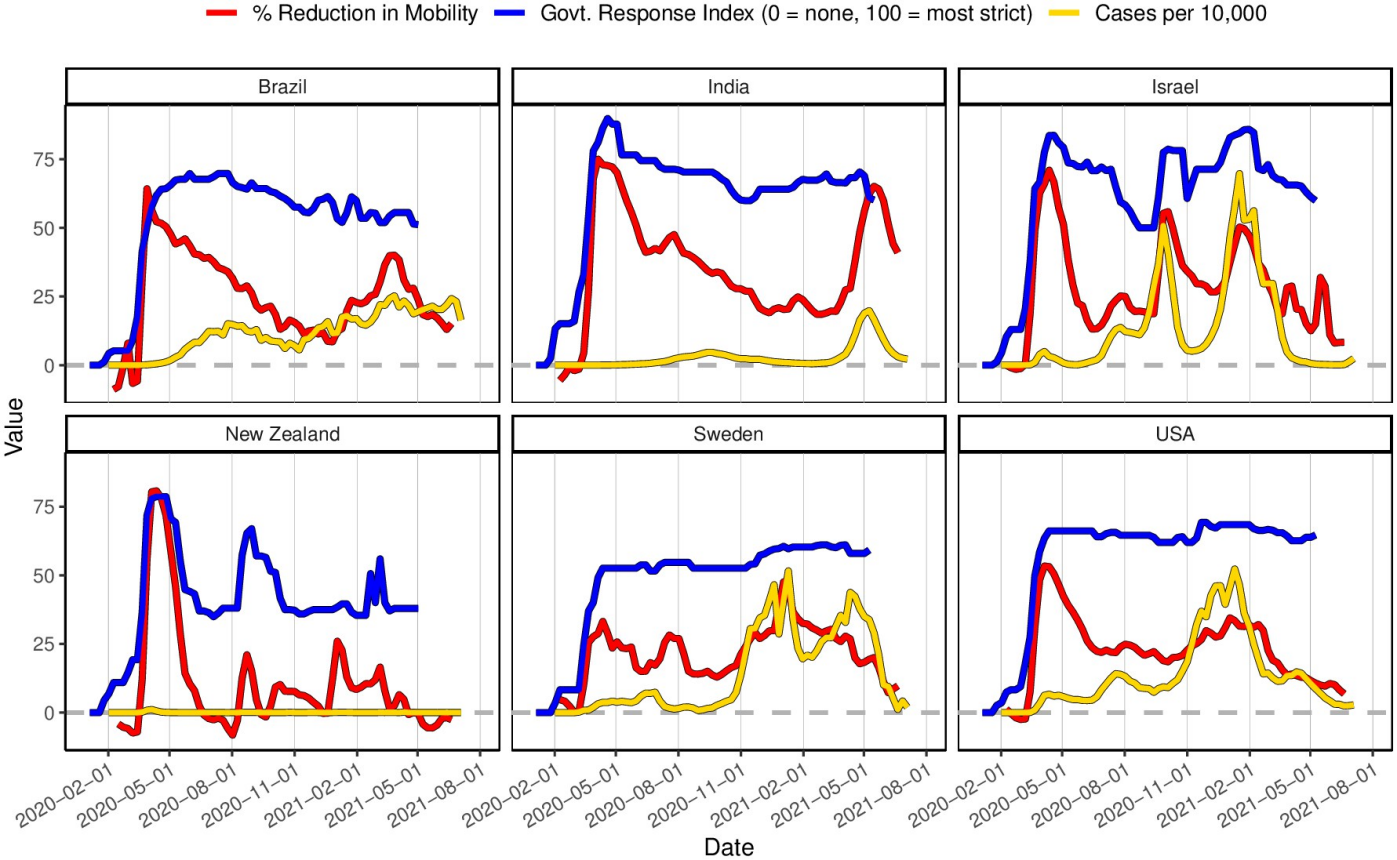
Relationship between COVID cases, % reduction in mobility, and governmental response^1^. ^1^ COVID cases are often presented as confirmed cases per 100,000 people. Here, we present these numbers per 10,000 people to facilitate visual comparison with other metrics of interest.

We first explore the time trends of each resilience metric for each country. Fig 3 shows the confirmed COVID-19 cases per 10,000, the percent reduction in mobility (relative to Jan.-Feb. 2020 baseline levels), and the governmental response index (0 = no response, 100 = strictest response).

#### Governmental intervention

In all countries, a rapid increase in governmental policy intervention is apparent during the initial lockdown phase around March 2020, indicating a fast initial shock and response within all countries. After the initial lockdown, governmental policy strictness varied substantially between countries. For the USA and Sweden, the policy was fairly constant over time, while policy strictness fluctuated in New Zealand and Israel. For Israel in particular, the governmental response was closely linked to confirmed case counts, perhaps indicating that policy decisions were being informed by disease rates. Brazil and India both had a gradual loosening of policy restrictions over the course of 2020 and the first half of 2021. On average, Sweden had the lowest level of governmental policy strictness, consistent with their early-pandemic “herd immunity” approach [34].

#### Adherence to government orders

While governments can impose policy in an effort to reduce disease spread, population adherence to these policies is key. According to the cell phone mobility data, all countries experienced some sort of initial lockdown, although the level of mobility reduction in Sweden was low compared to the other countries. As with governmental policy, mobility levels fluctuated substantially throughout the pandemic for Israel and New Zealand. For Israel and New Zealand, governmental response and reduction in mobility also had a fairly strong correspondence, suggesting that the population was responsive to changing governmental mandates. In contrast to New Zealand and Israel, mobility levels for India and Brazil gradually increased throughout 2020 after initial lockdown despite comparatively small change in governmental policy. There was little correspondence between governmental policy and mobility data after the initial lockdown in the USA, indicating a disconnect between governmental policy and population adherence/response.

#### (Confirmed) Clinical disease outcomes

**Fig 4:**
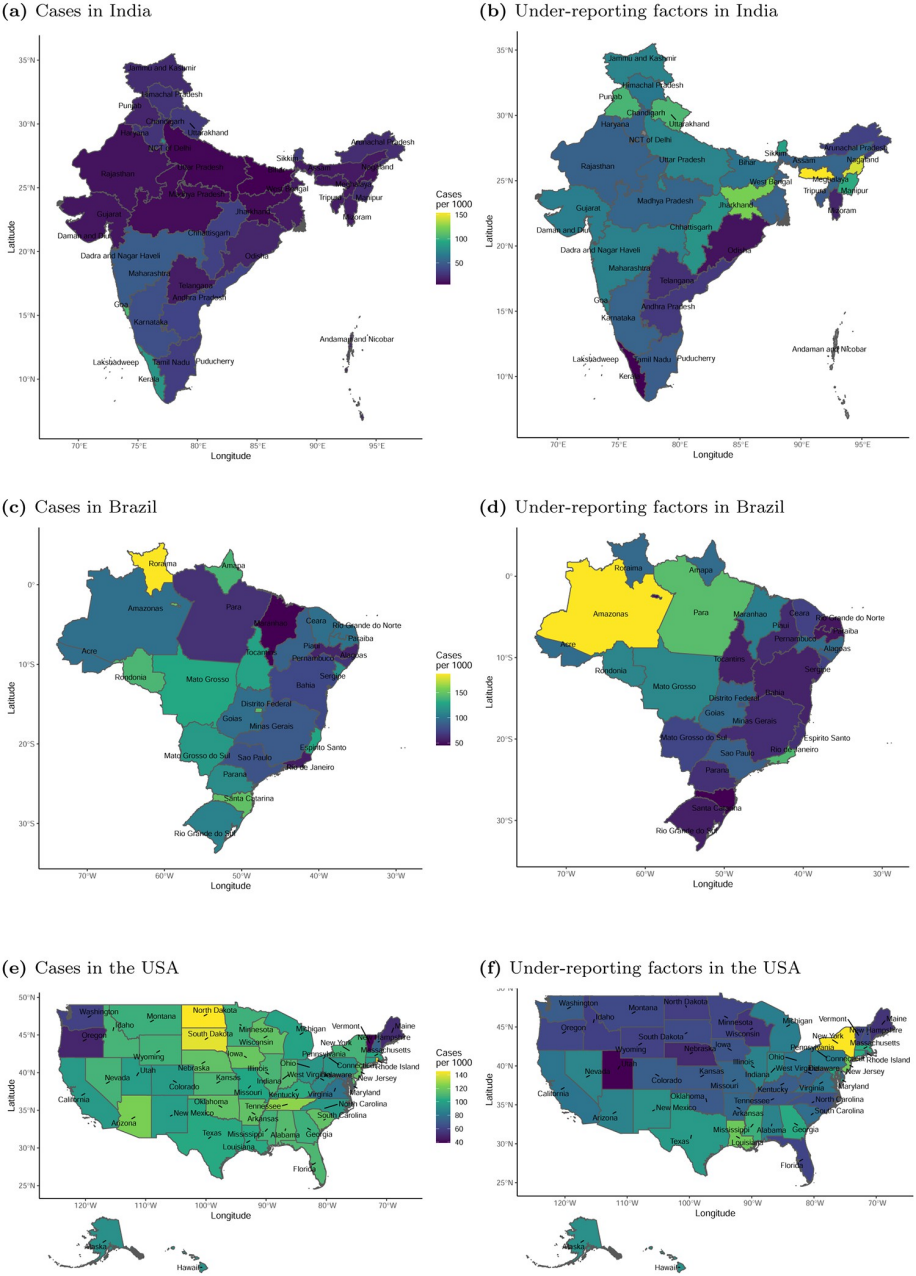
Confirmed COVID-19 cases and corresponding estimated cumulative under-reporting factors (URF) at the administrative 1 level for India, Brazil, and the USA^1^. (a) Cases in India, (b) Under-reporting factors in India, (c) Cases in Brazil, (d) Under-reporting factors in Brazil, (e) Cases in the USA, and (f) Under-reporting factors in the USA. ^1^ Corresponds to cumulative cases and corresponding under-reporting factors for the period under study, from February, 2020 through June, 2021. Under-reporting rates were adjusted for the population age distribution but not vaccination status.

New Zealand had very low case rates relative to the other countries, and Israel had comparatively high confirmed case rates relative to the size of the population. India and Brazil both reported comparatively lower *confirmed* case rates. However, it is important to interpret these case reports relative to the estimated amount of case under-reporting. For example, Brazil had a very high rate of COVID-related deaths (**S1 Fig C.1 in** S1 File), indicating a potentially large degree of under-reporting. Cumulative confirmed COVID-19 cases and corresponding estimated under-reporting factors were provided at the administrative 1 level for Brazil, India, and the USA in Fig 4.

#### Health care system/public health mobilization

Under-reporting (i.e., the estimated rate of true cases relative to those officially identified/reported) can be viewed as a metric of the performance of the public health response. This metric is important because identified cases are actionable whereas unidentified cases may result in enhanced disease spread. Using the method in **S1 Section B in** S1 File, we estimated the case under-reporting factors for each country over time. Results are shown in **S1 Fig C.2 in** S1 File. According to our estimates, New Zealand and Israel both had low rates of under-reporting, indicating that their public health response for identifying cases was more comprehensive than the other countries. In contrast, Brazil and India had very large estimated rates of under-reporting. This is consistent with testing levels in Brazil and India, which were extremely low relative to the other countries (**S1 Fig C.4 in** S1 File).

Another metric of health care mobilization is the rate of vaccination. Of course, we must interpret vaccination rates relative to vaccine availability, which was limited in most countries during the period under study. Vaccination rates as of July 2021 were very low in India, New Zealand, Brazil, and Sweden. Israel had comparatively high levels of vaccination, with over 60% of the population reported as being fully vaccinated. Over 40% of the USA population were reported as being fully vaccinated.

#### Political stability/protesting

**S1 Fig C.3 in** S1 File provides the total number of COVID-related battles, explosions, protests, riots, strategic developments, or violence against civilians recorded by ACLED during the course of the pandemic. Data for New Zealand were unavailable. Sweden had low levels of political instability as a direct result of the pandemic. In India and Brazil, protests, often in response to lockdown orders and masking requirements, occurred at high levels throughout 2020. Israel reported low levels of political unrest in the first half of 2020, but political backlash against corruption and handling of the COVID-19 pandemic resulted in mass protests in 2020 and early 2021 [16]. Protests against lockdowns and mask requirements erupted in the USA in the spring and summer of 2020 and continued through early 2021.

#### Economic outcomes

**S1 Fig C.5 in** S1 File shows the gross domestic product (GDP) and composite leading indicator (CLI) levels for each country over the course of the pandemic. Data for New Zealand were unavailable. All countries experienced an initial shock resulting in a drop in CLI and GDP levels, with India experiencing the greatest drop. This drop was the least severe for Sweden, possibly reflecting their less stringent governmental lockdowns. For all countries, there was a brief reduction in exports in early to mid-2020, with exports returning near to pre-pandemic levels toward the end of 2020. Both imports and exports were most strongly impacted in India in early 2020. Brazil and Sweden had a notable increase in imports relative to pre-pandemic levels in 2021. We discuss economic impacts by sector (e.g., manufacturing, hospitality, etc.) in **Section 4.4 in** S1 File.

#### Pre-COVID infectious disease vulnerability indices in Moore et al. (2016) [6]

[6] developed expert-informed indices predicting infectious disease pandemic vulnerability/resilience for countries across the globe. Overall metrics (0 = worst, 1 = best) for the six countries under investigation were as follows: USA (0.92), Brazil (0.72), India (0.49), New Zealand (0.92), Sweden (0.96), Israel (0.78). These scores predict greater pandemic resilience for the USA, New Zealand, and Sweden, moderate resilience for Brazil and Israel, and much lower resilience for India. Resilience metrics in Moore et al. (2016) [6] were also broken down by domains of societal resilience (e.g., economic, political). These indices are presented in Table 1b. India’s low aggregate score from Moore et al. (2016) [6] was due to low resilience scores across all dimensions, while lower scores in Israel were partially driven by lower domestic and international political resilience.

**Table 1:** Comparison of (a) our data-driven resilience rankings across various dimensions with (b) expert-informed pre-COVID infectious disease vulnerability indices previously developed by Moore et al. (2016) [6]^1^.

**(a) Data-Driven Rankings:** COVID-19 resilience across 6 countries based on our data-driven approach^2^						
*Country*	*Clinical Outcomes*	*Governmental Orders*	*Adherence to Govt. Orders*	*Health Care Mobilization*	*Political Stability*	*Economic Fluctuations*
Brazil	6 (Worst)	5	6 (Worst)	6 (Worst)	3	4
India	5	3	4	5	4	6 (Worst)
Israel	2	2	2	1 (Best)	6 (Worst)	5
New Zealand	1 (Best)	1 (Best)	1 (Best)	2	1 (Best)	3
Sweden	3	6 (Worst)	5	4	2	1 (Best)
USA	4	4	3	3	5	2

**(b) Expert-Informed Indices:** Pre-COVID infectious disease vulnerability indices in Moore et al. (2016) [6]^1^								
*Country*	*Overall*	*Demographic*	*Health Care*	*Public Health*	*Disease Dynamics*	*Political Domestic*	*Political International*	*Economic*
Brazil	0.72	0.87	0.68	0.89	0.35	0.55	0.75	0.49
India	0.49	0.53	0.41	0.61	0.44	0.47	0.55	0.34
Israel	0.78	0.85	0.77	0.92	0.60	0.58	0.38	0.75
New Zealand	0.92	0.95	0.80	0.82	0.81	0.96	0.92	0.80
Sweden	0.96	0.95	0.76	0.94	0.79	0.95	0.75	0.86
USA	0.92	0.80	0.80	0.97	0.38	0.90	0.92	0.88

^1^ Indices were calculated for 53 countries in Moore et al. (2016) [6] and standardized to a 0 (worst) to 1 (best) scale for each metric. Indices and corresponding rankings were based on expert opinion.

^2^ Clinical outcome rankings were based on under-reporting-corrected cases and deaths per 100,000 people rather than raw case and death counts. Hospitalizations per 100,000 were also considered.

- Governmental order rankings were based on the responsiveness of the governmental orders to higher or lower case confirmed counts; these are not an evaluation of the reasonableness of the governmental policy overall. An exception was made for Sweden, which had a much looser initial lockdown than the other countries.

- Rankings of adherence to government orders were based on the relationship between governmental order strictness and mobility data.

- Health care mobilization rankings were based on estimated case under-reporting rates, testing rates, and vaccination rates.

- Political stability rankings were based on the number of ACLED-recorded violence/protest/etc. events. News reports were used to evaluate the political climate in New Zealand.

- Economic fluctuations rankings were based on GDP/imports/exports fluctuations and speed of recovery.

We used insights from our visual assessment of data-driven resilience metrics to construct rough rankings of country-level *observed* pandemic resilience across multiple dimensions in Fig 1. In terms of (1) clinical outcomes, (2) health care system mobilization, (3) responsive-ness of governmental policy, and (4) population adherence to governmental policy, Israel and New Zealand had the best observed pandemic resilience. In terms of political stability during the period of study, New Zealand had the lowest rate of protests per capita, while Israel had the highest rate of protests. Interestingly, the USA’s observed political backlash to lockdown and mask policy was not captured by the Moore et al. (2016) [6] political resilience metric, while high protests rates in Brazil, India, and Israel were consistent with their lower political resilience scores. Negative economic impacts of the pandemic in terms of GDP and imports/exports were strongest in India and mildest in Sweden, broadly consistent with the results in Moore et al. (2016) [6].

High estimated under-reporting rates in India and Brazil and low rates of testing through 2020 reflect a comparatively weak healthcare system mobilization. According to Moore et al. (2016) [6], however, Brazil was predicted to have higher health care system and public health resilience than India. These discrepancies suggest that Brazil may have had greater resources than India to combat the virus, but this did not translate into better pandemic outcomes.

### 4.2 Associations between resilience metrics

In this section, we take a deeper dive into associations between resilience metrics within countries. Explorations of this type can provide insight into the interplay between many competing factors impacting governmental policy, clinical outcomes, and population response.

**S1 Fig D.2 in** S1 File provides the Spearman cross-correlations for pairs of resilience-related metrics overall and stratified by calendar quarter. Interestingly, we find that all metrics were strongly correlated in quarter 1 of 2020, during which time all countries experienced some form of lockdown. Many of these correlations attenuated or even changed direction in quarter 2 of 2020, when lockdown fatigue and other factors resulted in increased mobility in many countries. This changepoint in terms of pandemic response indicates a shift in each country’s pandemic response after initial lockdown and demonstrates that these policy and response changes differ between countries.

**Fig 5:**
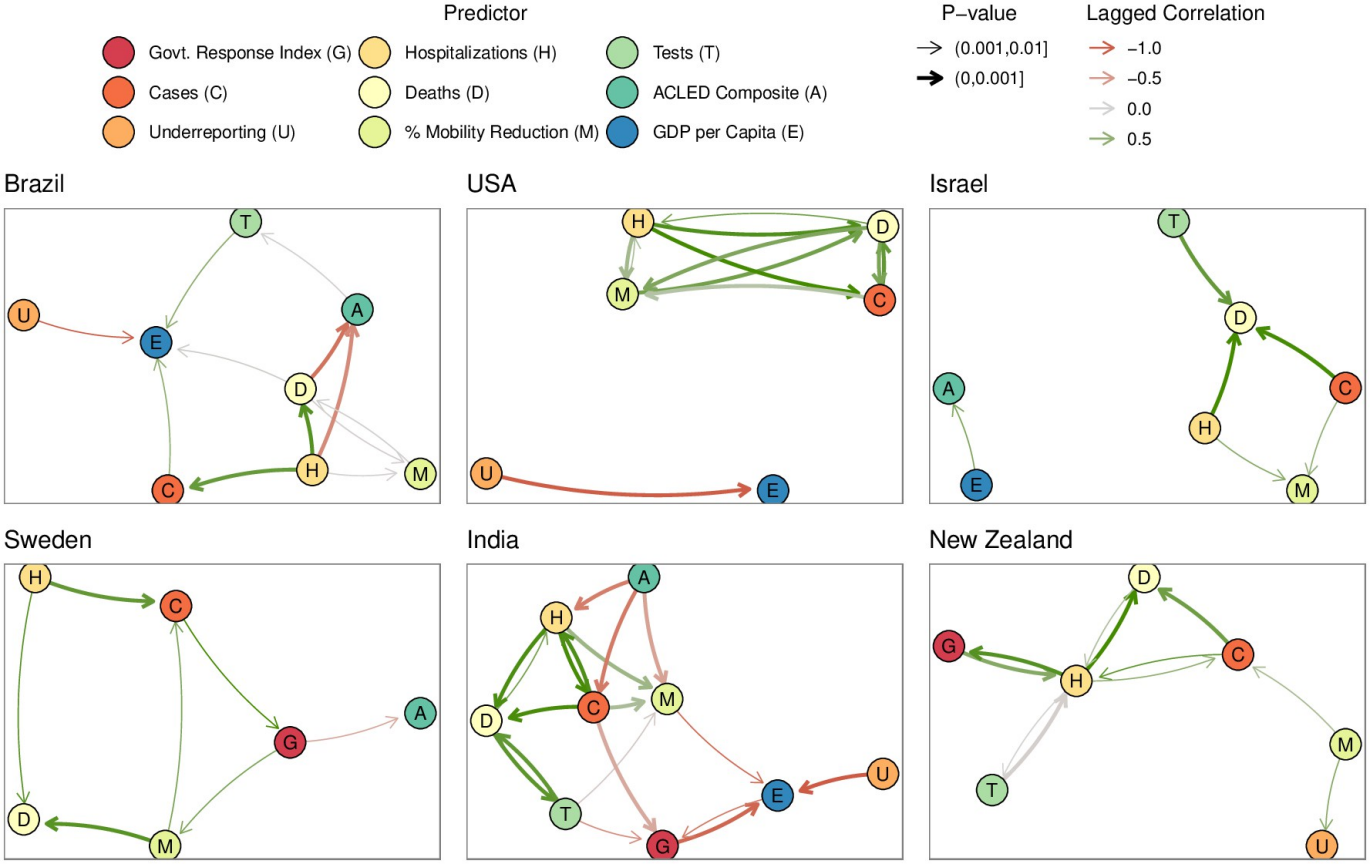
Network diagrams of time-lagged associations based on bivariate Granger association tests. ^1^ Tests significant at 0.01 are denoted by arrows. Tests are based on data for Quarter 2 in 2020 through Quarter 2 in 2021. For cases, deaths, hospitalizations, tests, ACLED composite (indicator of protests/unrest), and under-reporting factors, tests were based on log-transformed versions of the metrics to better satisfy normality assumptions. Arrow thickness and color correspond to the lagged Spearman cross-correlations of current values of one variable and the values of another variable two weeks ago. Arrows **should not be interpreted as causation** and instead represent time-lagged associations/correlations between pairs of time series.

The cross-correlations in **S1 Fig D.2 in** S1 File evaluate how metrics are correlated within the same week, but we may hypothesize that current resilience metrics such as governmental policy decisions could be informed by other metrics from the recent past (such as case rates and economic contraction). In **S1 Fig D.1 in** S1 File and Fig 5, we provide results from Granger association tests, which evaluate the time-related association between *past* values of a one metric (3 weeks prior) and *current* values of another metric, also adjusting for past values of the second metric. We emphasize that these results do not imply causality; confounding can certainly impact these associations. These associations, however, provide intuition into possible sequences of events. Past hospitalization rates were significantly associated with current death rates in all countries, which is expected due to the progression of disease in hospitalized patients. However, past official case counts were not significantly associated with future death rates for some countries, possibly due to confounding by under-reporting. Interestingly, higher estimates of case under-reporting were inversely associated with economic outcomes in Brazil, the USA, Sweden (p>0.01), and India. Higher past case counts were associated with a higher number of current protests in Israel.

### 4.3 Mixed modeling of relationships between resilience and pre-pandemic regional characteristics

Pairwise correlation analyses and Granger testing are useful for data exploration and developing intuition, but they have a large potential to be impacted by confounding. To (partially) address this, we estimate associations between resilience, adjusting for several pre-pandemic vulnerability indicators.

**Fig 6:**
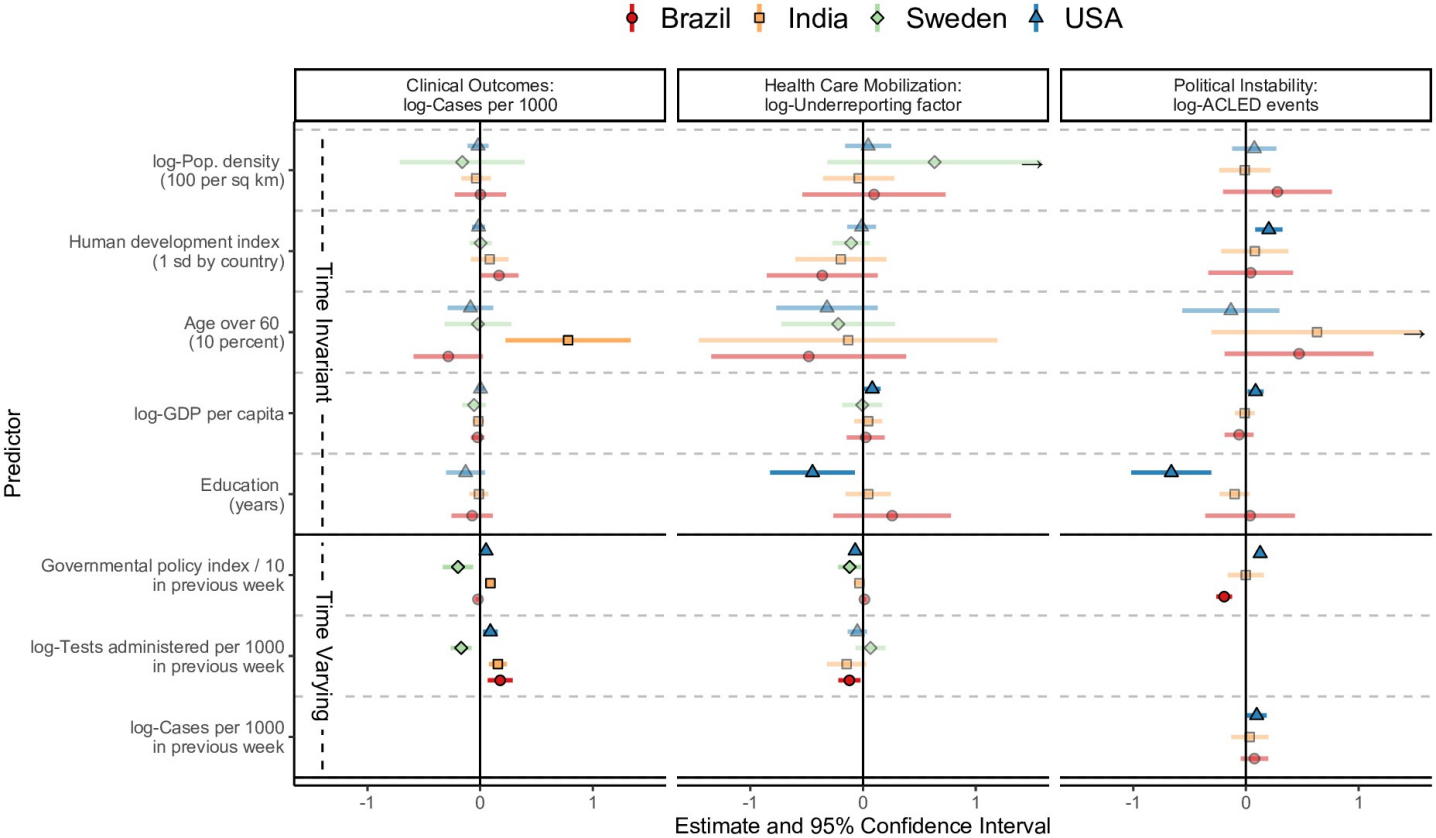
Associations from mixed modeling of pandemic outcomes over time at the regional level^1^. ^1^ Estimated parameters from linear mixed modeling of each resilience outcome for regions within each country. This mixed model included a random intercept for region and accounted for temporal correlation within each region through an auto-regressive moving average model for the errors. This model did not account for spatial correlation within each country. Six regions in India with missing covariates were excluded (out of 36).

Fig 6 shows the adjusted relationship between administrative level 1 (regional) pre-pandemic vulnerability characteristics and each of several region and time-varying resilience metrics, also adjusting for past values of other resilience metrics. Results for New Zealand and Israel were not included due to a lack of administrative level 1 data. We found that more educated US states (higher years of education on average) tended to have lower rates of case under-reporting and fewer COVID-related protests on average. Stricter prior-week COVID-related governmental policies were associated with higher numbers of protests in the US and a lower number of protests in Brazil. This reflects the cause of protesting, where US protests often were a response to enhanced governmental intervention, while Brazilian protests were often related to a lack of intervention (as recorded by ACLED and reported widely). Stricter prior-week governmental policy was also associated with lower rates of case under-reporting in the US and Sweden. While higher prior week testing rates per 1000 were associated with high case counts per 1000 in the US, India, and Brazil, prior week testing rates were inversely associated with case counts in Sweden. Testing rates were not significantly associated with estimated under-reporting rates in Sweden.

Among the pre-pandemic factors included in these models, state-level human development index, GDP, and education were significantly associated with resilience in the USA. Education in particular was not significantly associated with the resilience metrics in the other countries. Population density was not significantly associated with the number of cases per 1000 people for any of the four countries. Age distribution was strongly associated with case counts in India but not in Sweden or the USA.

The mixed modeling in Fig 6 does not account for the spatial structure within each country, where neighboring regions may tend to have higher correlation in case counts and other resilience metrics. In a sensitivity analysis focusing on the case counts outcome, we fit spatio-temporal Bayesian linear mixed models, accounting for the spatial structure within each country. We accounted for the spatial structure through an adjacency matrix that induced correlations between neighboring/bordering regions, and temporal correlations were again modeled using a simple autoregressive structure. Education was not included as a covariate in this model due to missing covariate information in India.

**S1 Table E.1 in** S1 File presents the spatio-temporal mixed model results for each country. We found that the strong association between age and log-cases for India seen in Fig 6 was no longer statistically significant when we accounted for spatial correlation (0.069, 95% CI [-0.10, 0.23]). In this model, the spatial and temporal correlation was large, causing a decrease in the estimated associations with age.

### 4.4 Predicted impact of pandemic on different economic sectors

**Fig 7:**
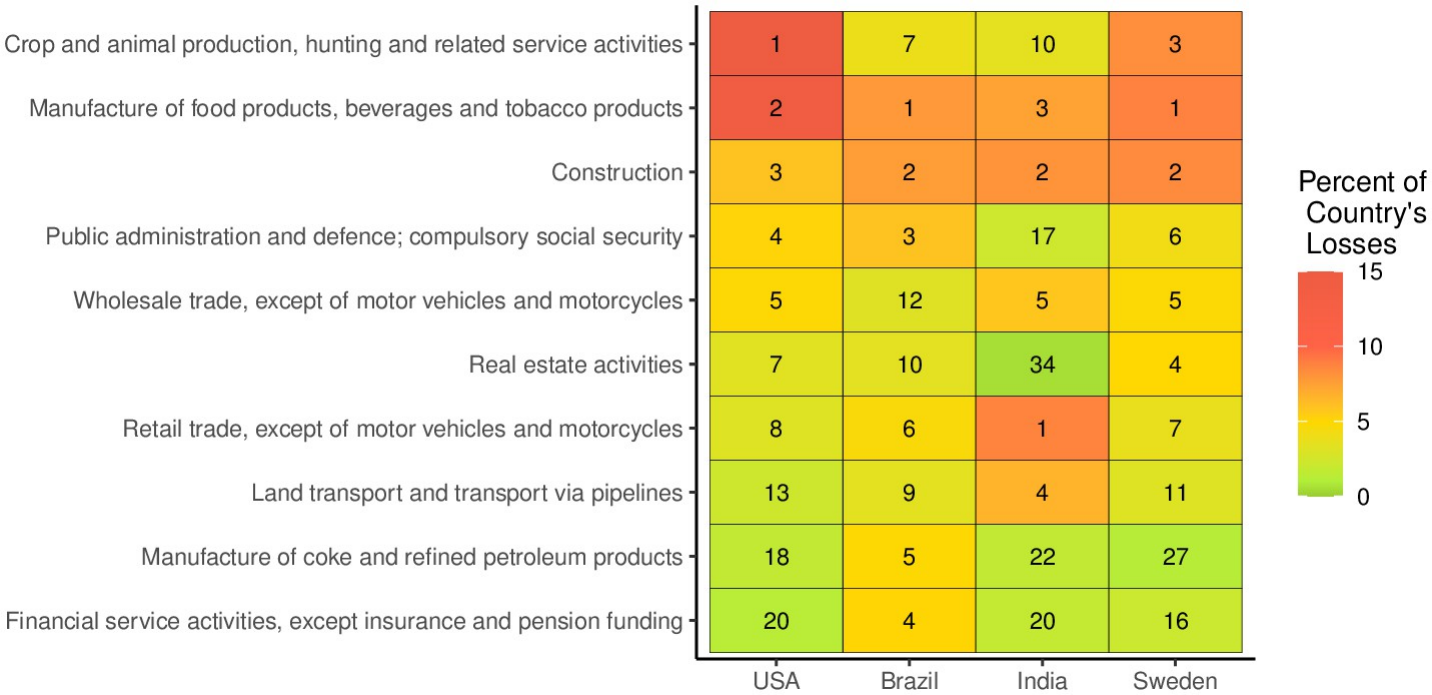
Top ranking sectors in terms of COVID-19 pandemic losses for each country, relative to total losses per country. ^1^Reported rankings are calculated relative to a total of 56 sectors for each country and include all sectors ranking in the top 5 for any of the four countries. Losses refer to national total losses in dollars and are presented as a percent of each country’s total losses.

In this section, we provide additional results relating to the pandemic’s impact on each country, stratified by economic sector. While there are many ways in which a pandemic can impact a national economy, this analysis estimates the percent of a country’s financial losses attributable to each sector due to a pandemic-related reduction in workforce. Fig 7 provides these results for the top five most-impacted industries for each country (11 industries total), where we define the level of impact in terms of the total national loss in dollars. New Zealand and Israel were excluded from this analysis since their input-output data tables were not available. For additional methods details and results, see **S1 Section F.3 in** S1 File and **S1 Fig F.4 in** S1 File.

Across all countries, manufacturing of food products and construction were among the sectors most strongly impacted by the pandemic. This is partly due to a high potential for occupation-related COVID exposure. Crop and animal production were also very strongly impacted in the USA and Sweden, while these sectors had a lesser relative pandemic impact in Brazil and India. Unlike the other countries, retail trade (excluding motor vehicles and motorcycles) was very strongly impacted in India relative to other sectors, as was the land transport sector. Differences between our estimated impacts of each sector between countries can be explained by three country characteristics: (1) the absolute importance of the economic sector in each country, (2) the assumed labor intensity (i.e., the proportion of labor per unit of capital) for each country, (3) the structure of the interconnection between sectors captured by the technical coefficient of the input-output model, and (4) the likelihood of COVID-19 exposure across sectors.

## 5 Discussion and conclusions

In this work, we describe a quantitative, data-driven approach to studying vulnerability and resilience across a variety of social, political, and economic domains and apply this approach to characterize resilience to the COVID-19 pandemic resilience in six countries. The COVID-19 pandemic provides a rich data ecosystem in which to study resilience, where key indicators were measured consistently across time for six countries. This allowed us to evaluate the impact of the *same* shock across multiple countries in a systematic and coherent way. This work demonstrates a quantitative vulnerability-resilience framework that can be applied to study resilience to incident disasters and other crises in the future, leveraging and increasingly diverse and rich data landscape.

Prior work studying vulnerability and resilience has primarily focused on predicting hypothetical resilience rather than quantifying observed resilience. Historically, the key limitation to data-driven, quantified analyses of resilience has been a lack of data itself. With its strong and pervasive impact globally, COVID-19 has led to the development of new data infrastructure and dissemination pipelines. Key indicators such as COVID-19 diagnoses are available at a fine spatial scale and collected across a wide breadth of locations. Additionally, a large number of indicators have been collected, allowing evaluation of resilience across many societal domains (e.g., economic, policy, etc). The public health and research communities have undergone a strong effort to ensure consistency in these data indicators globally, allowing data from different countries to be more directly comparable. Ultimately, the availability, quality, consistency, depth, and breadth of data available during the COVID-19 pandemic has provided an unprecedented opportunity to study resiliency in a holistic, data-driven manner. While the COVID-19 pandemic and corresponding data streams will not continue forever, the data infrastructure developed during this time period will be crucial to data-driven evaluation of resiliency and vulnerability moving forward.

Resilience in the context of the COVID-19 pandemic has been explored in other recent works. Brada et al. (2021) [9] explores resilience and recovery as a function of GDP and unemployment fluctuations throughout the pandemic in Central and Eastern Europe, exploring temporal and spatial associations with population density and other regional characteristics. Cartaxo et al. (2021) [5] identified three countries each with “good” and “bad” clinical outcomes. In both of these works, the impact of the pandemic was quantified using observed data in a *single dimension* (economic outcomes for Brada et al. (2021) [9] and clinical outcomes for Cartaxo et al. (2021) [5]). In contrast, our analysis explores resilience across many dimensions, taking a more holistic approach. Bloomberg’s COVID resilience ranking aggregates clinical, policy, economic, and mobility data to characterize resilience over time across many countries worldwide [35]. A severe limitation of this scoring system, however, is that it is a seemingly arbitrary composite of these disparate resilience measures, implicitly reflecting prioritization between different aspects of societal well-being.

In our exploration of multi-dimensional resilience to the COVID-19 pandemic, we integrate data on clinical disease dynamics, testing and vaccination administration, cell-phone-based mobility data, governmental policy, protest and violent event rates, and other data streams to characterize the impact of the pandemic, assess the relationships between resilience indicators across social and political domains, and evaluate their associations with pre-pandemic societal characteristics or vulnerabilities. This analysis provides insight into the impact of the pandemic on different facets of society and highlights pre-pandemic vulnerabilities associated with variation in pandemic resilience within each country.

During the period under study (February 2020 through roughly July 2021), we found that Brazil had the worst pandemic outcomes and response, particularly in terms of health care mobilization, clinical outcomes, appropriateness of governmental orders, and adherence to governmental orders. New Zealand had the best pandemic response overall, followed by Israel. The looser lockdown strategy taken by Sweden early in the pandemic did result in less severe economic shocks in terms of GDP reduction, but clinical outcomes and health care mobilization were poor compared to many of the other countries.

We found that the relationships between different domains of resilience (e.g., political, economic, etc.) were often different during and after initial lockdowns, where there was more heterogeneity in country response after initial lockdowns. We also observed differences between countries in terms of the direction of vulnerability-adjusted relationships between resilience indicators, where stricter governmental policy was associated with higher political unrest across states within the USA while the opposite was true for states in Brazil. Even after adjusting for strictness of governmental COVID policies, we found that higher education was significantly associated with lower amounts of political unrest across states in the USA. Heterogeneity in responses even within countries highlights the potential for regional differences, including education, to impact pandemic response. There was also heterogeneity in terms of the most pandemic-impacted sectors across countries. Food/beverage manufacturing and construction were strongly impacted relative to other sectors across all countries, while crop and animal production was less strongly impacted relative to other sectors in Brazil and India.

While this work provides insight into multi-domain resilience, future explorations should incorporate data from a wider array of countries and evaluate associations between observed resilience and a wider spectrum of potential geographic and societal vulnerabilities. Limited data availability and the potential for unmeasured confounding *severely limit our ability to make causal conclusions* from these analyses; rather, these results provide intuition about associations between the various data streams across time and space. Strong correlations over time between the variables of interest further limit our ability to make causal statements using these data. Additionally, imperfect data proxies were used to represent abstract concepts of resilience, and other key dimensions of societal resilience such as individual mental health, hunger, corruption, and well-being are not captured. Future work can implement a factor-based approach to understanding these abstract dimensions of resilience using multiple data proxies for each dimension, which may provide a more robust characterization of each conceptual resilience domain. Other limitations include the lack of adjustment for time since vaccination or booster status in the estimation of under-reporting factors and also arguably unrealistic assumptions used in the economic analysis by sector, such as the assumption that workers are unable to work for 3 weeks upon infection, regardless of employment sector.

Even in light of these limitations, these types of data-driven analyses have the potential to reveal insights and guide intuition about the interplay between resiliency and vulnerability. Below, we summarize several key takeaways from our exploration that can guide vulnerability-resilience analyses in the future:

1. Multiple metrics of resilience are needed to capture resilience holistically. Analysis of economic indicators alone may provide an incomplete picture of resilience, and many key factors related to country pandemic response such as political polarization or backlash may not be fully captured (Table 1). Efforts to construct a single metric of resilience or vulnerability make implicit assumptions about the prioritization of various aspects of societal well-being and should be used with extreme caution.
2. Intra-country heterogeneity can be substantial (especially for large countries), and variation in pre-pandemic vulnerabilities may be associated with differences in observed resilience metrics even within countries. This motivates country-specific analysis in the future, suggesting that we should not take a one-size-fits-all approach for characterizing a country’s resilience. (Figs 6 and 7).
3. Prior work predicting resiliency characterized resource availability but did not consider the possibility that countries may *choose not to* mobilize those resources when faced with a crisis, possibly in response to misinformation, crisis denialism, and fears of political backlash. Additionally, governmental policy restrictions may not always be followed by the population (Fig 3). More research is need to disentangle the complicated interplay between resource availability, governmental response, population adherence, and political backlash. Existing literature studying individuals’ behavior and decisions when faced with infectious diseases could guide this work [36–39].
4. Attitudes and governmental policy may shift during the course of a crisis, and our analysis demonstrated that country responses differed during the initial shock phase and subsequent disease waves throughout the course of the pandemic (e.g., Fig 3). This motivates future exploration into governmental and population attitudes and interventions during different phases of a crisis.
5. Heterogeneous data and data fusion approaches are needed to understand and quantify trends, impacts, and dynamics during a crisis. The coronavirus pandemic gave rise to a new data revolution and as such, many data sources such as mobility indicators, daily clinical surveillance data, and hospitalizations became available. These types of data streams are needed to implement data-driven approaches that can help develop strategies to better address and respond to future crises.

In an increasingly data-rich world, there is a huge potential to harness a wide array of diverse data streams to gain insight into populations and their responses to disasters. Our results emphasize, however, that care must be taken to avoid over-simplifying subtleties and interactions between economic, political, and social responses within countries through use of single-indicator resilience metrics.

## Supporting information

S1 File (PDF)

## References

[1] Brenkert AL, Malone EL. Modeling vulnerability and resilience to climate change: A case study of India and Indian states. Climatic Change. 2005;72:57–102. doi: 10.1007/s10584-005-5930-3

[2] Malone EL. Vulnerability and Resilience in the Face of Climate Change: Current Research and Needs for Population Information; 2009.

[3] Kontokosta CE, Malik A. The Resilience to Emergencies and Disasters Index: Applying big data to benchmark and validate neighborhood resilience capacity. Sustainable Cities and Society. 2018;36:272–285. doi: 10.1016/j.scs.2017.10.025

[4] Angeon V, Bates S. Reviewing composite vulnerability and resilience indexes: A sustainable approach and application. World Development. 2015;72:140–162. doi: 10.1016/j.worlddev.2015.02.011

[5] Cartaxo ANS, Barbosa FIC, de Souza Bermejo PH, Moreira MF, Prata DN. The exposure risk to COVID-19 in most affected countries: A vulnerability assessment model. PLoS ONE. 2021;16(3):1–20. doi: 10.1371/journal.pone.0248075 33662028 PMC7932136

[6] Moore M, Gelfeld B, Okunogbe A, Paul C. Identifying Future Disease Hot Spots: Infectious Disease Vulnerability Index; 2016. 10.7249/rhq-06-03-05 PMC5568150 28845357

[7] Kimber LR. In: Wiig S, Fahlbruch B, editors. Resilience from the United Nations Standpoint: The Challenges of “Vagueness”. Cham: Springer International Publishing; 2019. p. 89–96. Available from: 10.1007/978-3-030-03189-3_11.

[8] Poljanšek K, Marin-Ferrer M, Vernaccini L, Messina L. Incorporating epidemics risk in the INFORM Global Risk Index; 2018.

[9] Brada JC, Gajewski P, Kutan AM. Economic resiliency and recovery, lessons from the financial crisis for the COVID-19 pandemic: A regional perspective from Central and Eastern Europe. International Review of Financial Analysis. 2021;74:1–12. doi: 10.1016/j.irfa.2021.101658 36567807 PMC9759986

[10] Nethery RC, Katz-Christy N, Kioumourtzoglou Ma, Parks RM, Schumacher A, Anderson GB. Integrated causal-predictive machine learning models for tropical cyclone epidemiology. Biostatistics. 2021; p. 1–16. 34962265 10.1093/biostatistics/kxab047 PMC10102905

[11] Diop S, Asongu SA, Nnanna J. COVID-19 economic vulnerability and resilience indexes: Global evidence. International Social Science Journal. 2021;71(S1):37–50. doi: 10.1111/issj.12276 34548690 PMC8447304

[12] United Nations Second Administrative Level Boundaries; 2021. Available from: https://www.unsalb.org/home.

[13] Badr HS, Zaitchik BF, Kerr GH, Nguyen NL, Chen YT, Hinson P, et al. Unified real-time environmental-epidemiological data for multiscale modeling of the COVID-19 pandemic. medRxiv. 2021. 10.1038/s41597-023-02276-y PMC10245354 37286690

[14] IHME. Data and Forecast Repository; 2021. Available from: http://www.healthdata.org/covid/data-downloads.

[15] Ritchie H, Mathieu E, Rodes-Guirao L, Appel C, Giattino C, Ortiz-Ospina E, et al. Coronavirus Pandemic (COVID-19). Our World in Data. 2020;.

[16] Clionadh R, Andrew L, Havard H, Joakim K. Introducing ACLED-Armed Conflict Location and Event Data. Journal of Peace Research. 2010;47(5):651–660. doi: 10.1177/0022343310378914

[17] Thomas H, Noam A, Rafael G, Beatriz K, Anna P, Toby P, et al. A global panel database of pandemic policies (Oxford COVID-19 Government Response Tracker). Nature Human Behaviour. 2021;5:529–538. doi: 10.1038/s41562-021-01079-8 33686204

[18] OECD. Composite leading indicator (CLI) (indicator). 2021.

[19] FRED. federal reserve economic data. St. Louis, MO: Federal Reserve Bank of St. Louis; 1997. Available from:https://lccn.loc.gov/98802805.

[20] WTO. World Trade Organization. International trade statistics; 2021. Available from: https://timeseries.wto.org.

[21] BLS. U.S. Bureau of Labor Statistics; 2021.

[22] Timmer MP, Dietzenbacher E, Los B, Stehrer R, de Vries GJ. An Illustrated User Guide to the World Input–Output Database: the Case of Global Automotive Production. Review of International Economics. 2015;23:575–605. doi: 10.1111/roie.12178

[23] Timmer MP, Los B, Stehrer R, de Vries GJ. An Anatomy of the Global Trade Slowdown based on the WIOD 2016 Release. GGDC research memorandum number 162. 2016;.

[24] CIESIN - Columbia University. Gridded Population of the World, Version 4 (GPWv4): Administrative Unit Center Points with Population Estimates; 2016. Available from:10.7927/H4F47M2C.

[25] Kummu M, Taka M, Guillaume JHA. Gridded global datasets for Gross Domestic Product and Human Development Index over 1990-2015. Scientific Data. 2018;5:1–15. doi: 10.1038/sdata.2018.4 29406518 PMC5800392

[26] Gorelick N, Hancher M, Dixon M, Ilyushchenko S, Thau D, Moore R. Google Earth Engine: Planetary-scale geospatial analysis for everyone. Remote Sensing of Environment. 2017;202:18–27. doi: 10.1016/j.rse.2017.06.031

[27] Global Administrative Areas v3.6; 2021. https://globaldatalab.org/shdi/msch/.

[28] Global Data Lab. Subnational SDG Dashboard; 2021. Available from: https://globaldatalab.org/shdi/msch/.

[29] Lau H, Khosrawipour T, Kocbach P, Ichii H, Bania J, Khosrawipour V. Evaluating the massive underreporting and undertesting of COVID-19 cases in multiple global epicenters. Pulmonology. 2020;27(2):110–115. doi: 10.1016/j.pulmoe.2020.05.015 32540223 PMC7275155

[30] O’Driscoll M, Ribeiro Dos Santos G, Wang L, Cummings DAT, Azman AS, Paireau J, et al. Age-specific mortality and immunity patterns of SARS-CoV-2. Nature. 2021;590(7844):140–145. doi: 10.1038/s41586-020-2918-0 33137809

[31] Lee D, Rushworth A, Napier G. Spatio-Temporal Areal Unit Modeling in R with Conditional Autoregressive Priors Using the CARBayesST Package. Journal of Statistical Software. 2018;84(9):1–39. doi: 10.18637/jss.v084.i0930450020

[32] Leontief W. Input-output Economics. OUP E-Books. Oxford University Press; 1986.

[33] Miller RE, Blair PD. Input-Output Analysis: Foundations and Extensions. Cambridge University Press; 2009. Available from: https://books.google.com/books?id=viHaAgAAQBAJ.

[34] Claeson M, Hanson S. COVID-19 and the Swedish enigma. The Lancet. 2020;397(10271):P259–261. doi: 10.1016/S0140-6736(20)32750-1 33357494 PMC7755568

[35] Chang R. Methodology: Inside Bloomberg’s Covid Resilience Ranking; 2020.

[36] Moran K, Del Valle S. A Meta-Analysis of the Association between Gender and Protective Behaviors in Response to Respiratory Epidemics and Pandemics. PLOS One. 2016;11(10):1–25. doi: 10.1371/journal.pone.0164541 27768704 PMC5074573

[37] Bootsma MCJ, Ferguson NM. The effect of public health measures on the 1918 influenza pandemic in U.S. cities. PNAS. 2007;104(18):7588–7593. doi: 10.1073/pnas.0611071104 17416677 PMC1849868

[38] Poletti P, Caprile B, Ajelli M, Merler S. In: Uncoordinated Human Responses During Epidemic Outbreaks. Springer Science+Business Media; 2013.

[39] Bedson J, Skrip LA, Pedi D, Abramowitz S, Carter S, Jalloh MF, et al. A review and agenda for integrated disease models including social and behavioural factors. Nature Human Behavior. 2021;5(1):834–846. doi: 10.1038/s41562-021-01136-2 34183799

