# Supplementary material for: Multi-dimensional resilience: A quantitative exploration of disease outcomes and economic, political, and social resilience to the COVID-19 pandemic in six countries
Source: PLoS One. 2023 Jan 5;18(1):e0279894. doi: 10.1371/journal.pone.0279894 (PMC9815572; doi:10.1371/journal.pone.0279894)
Supplement: S1 File — (PDF) [file pone.0279894.s001.pdf]

Supporting Information for "Multi-dimensional resilience: a quantitative exploration of disease outcomes and economic, political, and social resilience to the COVID-19 pandemic in six countries" by

Lauren J. Beesley[*1,2], **Paolo Patelli**[1], **Kimberly Kaufeld**[2], **Jon Schwenk**[3], **Kaitlyn M. Martinez**[1], **Travis Pitts**[4], **Martha Barnard**[1], **Ben McMahon**[5], and **Sara Y. Del Valle**[1]

[1]Information Systems & Modeling, Los Alamos National Laboratory, Los Alamos, New Mexico, United States of America
[2]Statistical Sciences, Los Alamos National Laboratory, Los Alamos, New Mexico, United States of America
[3]Earth Systems & Observations, Los Alamos National Laboratory, Los Alamos, New Mexico, United States of America
[4]Intelligence & Systems Analysis, Los Alamos National Laboratory, Los Alamos, New Mexico, United States of America
[5]Theoretical Biology & Biophysics, Los Alamos National Laboratory, Los Alamos, New Mexico, United States of America

*Corresponding Author: lvandervort@lanl.gov

Contents

A **Data sources** **2**

B **Estimating case under-reporting based on case fatality rates** **5**

C **Visualizations of resilience over time** **9**

D **Associations between resilience metrics** **13**

E **Additional modeling results** **15**

F **Economic analysis** **16**

A Data sources

In this section, we provide additional information about the data sources and data resolutions used. **Table A.1** provides accession information for the data, including website links. **Table A.2** provides a catalog of the time and spatial resolution of each of these variables in each of the countries. Although not shown here, some data sources were available at further granular resolutions (e.g., daily cases or county-level data), but all data sources were aggregated up to the weekly and administrative level 1 resolutions. Resolutions in **Table A.2** correspond to the aggregated versions of the data used in this analysis. Temporal coverage of each time-varying variable are provided for each country in **Fig A.1**.

Table A.1. Information about data sources

Data Source	Variables	Access Date	Time Coverage[1]	Ref
John's Hopkins CSSEGIS	Cases	07-08-2021	02/2020 to 07/2021	[1]
	Deaths		03/2020 to 07/2021	
	Population density		Pre-pandemic	
Armed Conflict Location & Event Data Project (ACLED)	Number of Conflicts/Protests	04-12-2021	12/2019 to 03/2021	[2]
Our World in Data	People fully vaccinated	07-08-2021	01/2021 to 06/2021	[3]
OxCGRT	Governmental response indices	05-10-2021	01/2020 to 05/2021	[4]
Institute for Health	Predicted hospital admissions	07-12-2021	02/2020 to 07/2021	[5]
Metrics and Evaluation	Mobility composite		02/2020 to 06/2021	
(IHME)	Tests administered		02/2020 to 06/2021	
	ICU bed capacity		Pre-pandemic	
Organisation for Economic	Composite leading indicator	07-29-2021	12/2019 to 06/2021	[6]
Co-operation and Development (OECD)	Country-level GDP		12/2019 to 02/2021	
World Trade Organization	Exports	08-03-2021	12/2019 to 06/2021	[7]
(WTO)	Imports		12/2019 to 05/2021	
Global Data Lab	Years of schooling	08-13-2021	Pre-pandemic	[8]
Kummu et al. (2018)	Pre-pandemic GDP per capita	05-20-2021	Pre-pandemic	[9]
	Human development index		Pre-pandemic	
Gridded Population of the World	Age distributions	6-16-2021	Pre-pandemic	[10]
US Bureau of Labor Statistics (BLS)	US unemployment data	08-03-2021	01/2010 to 06/2021	[11]
World Input-Output Database (WIOD)	Input-Output tables	6-16-2021	Pre-pandemic, 2016 release	[12]

[1] Time coverage corresponds to the longest time period with available data for any country. "Pre-pandemic" indicates a time-invariant variable collected for some pre-pandemic time.

Table A.2. Summary of data availability and resolutions[1]

Variable	Time	USA	Brazil	NZ	India	Israel	Sweden
**Demographics and other regional characteristics**							
Population	-	Adm1	Adm1	Adm0	Adm1	Adm0	Adm1
Age distribution	-	Adm1	Adm1	Adm1[2]	Adm1	Adm1	Adm1
Population density	-	Adm1	Adm1	Adm0	Adm1[2]	Adm0	Adm1
Human development index, 2015	-	Adm1	Adm1	Adm1	Adm1	Adm1	Adm1
ICU bed capacity	-	Adm1[2]	Adm1	Adm0	-	Adm0	Adm0
Years of education, 2019	-	Adm1	Adm1	Adm1[2]	Adm1[2]	Adm0	Adm0
**Clinical disease outcomes**							
COVID cases	Weekly	Adm1	Adm1	Adm0	Adm1	Adm0	Adm1
COVID deaths	Weekly	Adm1	Adm1	Adm0	Adm1	Adm0	Adm1
People fully vaccinated	Weekly	Adm0	Adm0	Adm0	Adm0	Adm0	Adm0
Tests administered	Weekly	Adm1	Adm1	Adm0	Adm1	Adm0	Adm0
Predicted hospital admissions	Weekly	Adm1	Adm1	Adm0	Adm1	Adm0	Adm0
**Economic indicators**							
Gross domestic product per capita, 2015	-	Adm1	Adm1	Adm1	Adm1	Adm1	Adm1
Gross domestic product, de-trended	Monthly	Adm1	Adm0	-	Adm0	Adm0	Adm0
Composite leading indicator	Monthly	Adm1	Adm0	-	Adm0	Adm0	Adm0
Imports (millions of dollars)	Monthly	Adm0	Adm0	Adm0	Adm0	Adm0	Adm0
Exports (millions of dollars)	Monthly	Adm0	Adm0	-	Adm0	Adm0	Adm0
**Other indices of interest**							
Cell phone-based mobility composite	Weekly	Adm1	Adm1	Adm0	Adm1	Adm0	Adm0
COVID-related governmental policy	Weekly	Adm1	Adm1	Adm0	Adm0	Adm0	Adm0
Number of violent events/protests	Weekly	Adm1	Adm1	-	Adm1	Adm1	Adm1

[1] Additional details about data sources can be found in **Table A.1**. 'Adm0' corresponds to (country-level) administrative level 0 resolution, and 'Adm1' corresponds to more granular (regional) administrative level 1 resolution.
[2] Data are missing for at least one region.

Table A.3. Summary of pre-pandemic country characteristics [1]

Characteristic	Brazil	India	Israel	New Zealand	Sweden	USA
Regions Included	27	36	7	19	21	51
Population	212,559,409	1,380,004,385	8,655,541	4,822,233	10,099,270	329,466,283
Pre-pandemic ICU bed capacity	15,144	-	429	267	1,077	70,098
Pre-pandemic GDP per capita						
Overall	854,494	12,139,192	17,115,270	949,754	3,276,744	4,457,230
Maximum of regions	46,653,893	156,160,619	179,843,396	11,572,468	19,242,806	231,731,528
Minimum of regions	14,677	263,778	491,854	13,968	559,662	42,873
Population density						
Overall	21.3	357.9	297.7	10.5	9.1	17.3
Maximum of regions	458.2	9,720.2	-	-	148.8	2,507.0
Minimum of regions	2.4	16.2	-	-	0.8	0.009
Missing region values	0	1	0	0	0	0
Years of schooling						
Overall	8.0	6.5	13.0	12.8	12.5	13.4
Maximum of regions	10.4	10.1	-	13.5	-	14.5
Minimum of regions	6.2	4.6	-	11.7	-	12.9
Missing region values	0	5	All	6	All	0
Percent over 60						
Overall	10.7	8.6	11.9	16.5	24.9	18.4
Maximum of regions	13.6	12.6	19.3	27.9	30.0	23.1
Minimum of regions	5.1	4.6	8.1	16.3	20.6	12.7
Missing region values	0	0	0	1	0	0
Human development index						
Overall	0.72	0.64	0.89	0.91	0.90	0.91
Maximum of regions	0.86	0.99	0.90	0.92	0.94	0.96
Minimum of regions	0.66	0.49	0.87	0.89	0.89	0.79

[1] Maximum and minimum correspond to values across regions within each country.

Figure A.1. Time period of data availability for each country [1]

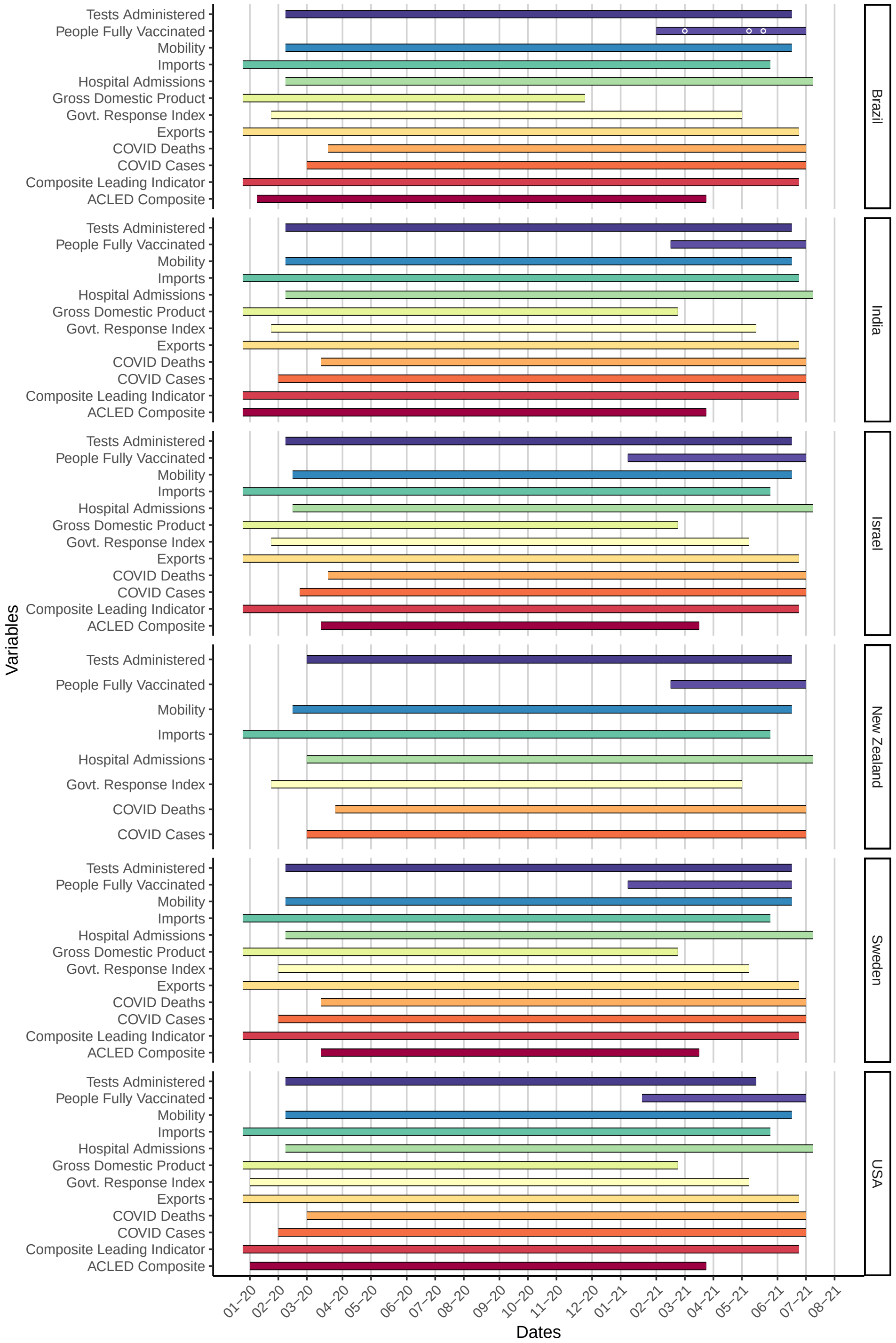

[1] Missing vaccination data for Brazil are denoted with white circles. The left-hand interval start location captures either 1) the earliest date with available data or 2) the first date with non-null values such as time prior to the first confirmed COVID-19 case or time prior to vaccine availability.

B Estimating case under-reporting based on case fatality rates

In this section, we describe the strategy used to estimate COVID case under-reporting in the main paper. We used a modification of the method proposed in Lau et al. (2020) [13].

Lau et al. (2020) [13] proposes estimating under-reporting based on the comparison between the observed case-fatality rate (CFR) among reported COVID-19 cases and an assumed "true" infection fatality rate (IFR) among all infected individuals. The first challenge is in estimating the case fatality rate in the observed data, and the second challenge is in defining the "true" infection fatality rate.

Several authors including Lau et al. (2020) [13] propose estimating the CFR as the total deaths by week w as a proportion of the total cases by week w-2. The goal of the time lag in deaths is to partially account for the delay in deaths among people diagnosed with COVID-19 in a given week. This strategy provides an estimated CFR over the entire course of the pandemic. However, since testing practices changed dramatically across the pandemic and our interest is in the under-reporting in a given time period rather than overall across the entire pandemic, we propose an alternative definition of the CFR within the last month as follows:

$$CFRrolling(w) = \frac{\text{number of deaths between week w and w-4}}{\text{number of cases between week w-2 and w-6}}, \qquad (\textit{Eq. S1})$$

where $CFRrolling(w)$ was then smoothed across 3 week intervals. The proposed rolling monthly CFR estimates were designed to be used for correcting incident cases for under-reporting rather than to correct cumulative cases, as described later on.

After we calculate the CFR, we need to define the target IFR that will serve as the assumed "truth" for each administrative level 1 region. We will assume the true IFR by age follows values reported in O'Driscoll et al. (2021) [14] based on meta-analysis and ensemble modeling across countries. These target IFR values by age are presented in **S1 Fig B.1**.

For each country or region, we compute the age-adjusted target IFR as the weighted average of the O'Driscoll et al. (2021) [14] IFR estimates based on the region's age distribution. This adjustment step is critical, since the age distributions can vary substantially between regions within each country and between countries on average. For example, India tends to be younger and Sweden tends to be older on average, relative to the other countries. The administrative level 1 target IFR values are presented in **S1 Fig B.2**, and national values were also calculated.

Using these target IFRs and the $CFRrolling(w)$ estimates, we then calculate the adjusted case counts using the following formulas, where the multiplicative factors is called the under-reporting factor (URF):

$$\text{Adjusted Incident Cases}(w) = \text{new cases for week w} * \frac{\text{smoothed}(CFRrolling(w))}{\text{age-adjusted target IFR}} \qquad (\textit{Eq. S2})$$

A key assumption of this approach is that it assumes no over-reporting of deaths. However, this would mean that the CFRs would be under-estimates and the corresponding under-reporting factors would also be under-estimates. So the adjusted cases would under-estimate the true cases. Additionally, this analysis defines the target IFR adjusting for age but not adjusting for other country-level differences, including comorbidities and access to healthcare. Finally, the target IFR by age provided by O'Driscoll et al. (2021) [14] was estimated in unvaccinated individuals, and this IFR may be different among vaccinated individuals. This is not accounted-for in our

estimates of the degree of under-reporting and may result in inaccurate estimates of under-reporting factors for more recent time-periods with greater vaccination rates.

Figure B.1. Target IFR by age reported in O'Driscoll et al. (2021) [14]

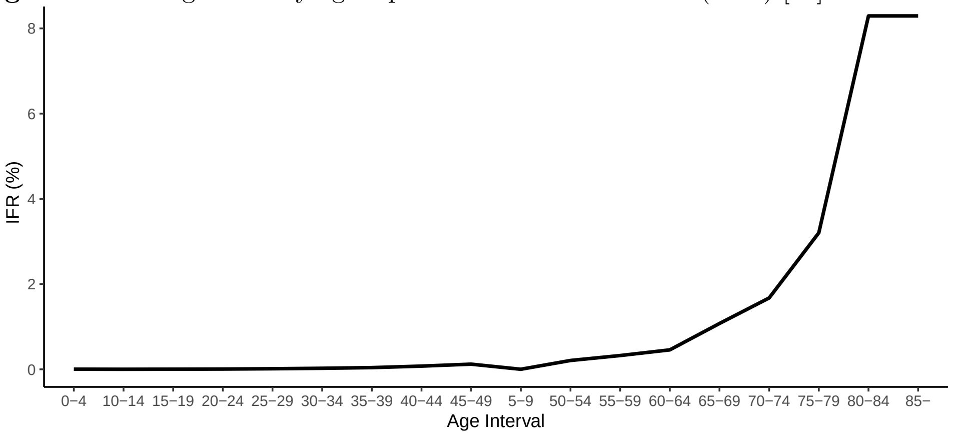

Figure B.2. Distributions of age-adjusted target IFRs by country [1]

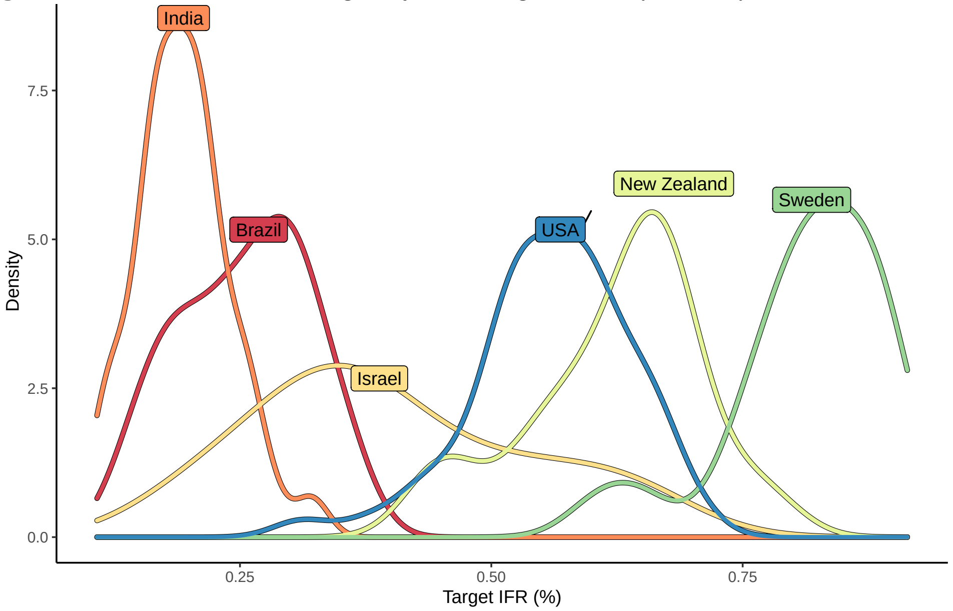

[1] Distributions correspond to age-adjusted target IFR values across regions within each country

B.1 Extension to incorporate vaccination information

We hypothesize that rate of vaccination in the population will have an impact on the "target" IFR, even adjusting for age, and the method proposed in *Eq. S2* will not incorporate this. The complicated relationship between vaccination may be related to the time since the last vaccination, receipt of a booster, the type of circulating COVID variant, etc. Therefore, it is not trivial to fully account for the impacts of vaccination on the target IFR.

As a rough sensitivity analysis, however, we propose a method for estimating under-reporting rates, assuming different simple relationships between vaccination and

IFR. In particular, suppose vaccination impacts the likelihood of death among infected individuals multiplicatively, i.e., the IFR among vaccinated individuals is θ times the IFR among unvaccinated individuals. Here, we define vaccinated individuals as individuals reported to by fully vaccinated, with or without a booster. Then, we could calculate a rough vaccination-adjusted IFR estimate using the following equation:

$$IFR_{new} = \theta * IFR * p_{vax} + IFR * (1 - p_{vax}), \qquad (Eq.\ S3)$$

where p_{vax} is the proportion of the population that is fully vaccinated, IFR refers to the age-adjusted infection fatality ratio for unvaccinated individuals based on estimates from O'Driscoll et al. (2021) [14], and $\theta \in (0, 1]$ is a sensitivity parameter controlling the overall impact of vaccination on the IFR. Then, we obtain updated estimates of the under-reporting factors by implementing the method in *Eq. S2* using the new vaccination- and age-adjusted estimate of the IFR.

Fig B.3 shows estimated under-reporting factors assuming different values of θ for the first half of 2021. Unsurprisingly, accounting for vaccination has the most impact for countries with the highest rates of vaccination during this time period, as shown in **Fig C.4**. Accounting for vaccination status *increases* the corresponding under-reporting estimates, since vaccinated individuals are expected to have similar or lower infection fatality rates relative to unvaccinated individuals. Therefore, the under-reporting estimated by ignoring vaccination status may often be a conservative (biased low) estimate of the true amount of under-reporting.

We also notice that Sweden has estimated under-reporting factors less than 1 during the spring/summer of 2021, as illustrated by **Fig B.3**. This indicates observed case rates that are *higher* than expected, relative to the observed death rates. One possible explanation for this is that COVID-related deaths may be under-reported. However, this death under-reporting would need to be substantial to explain estimated under-reporting factors of around 0.5. An alternative explanation is that the target IFR values used in this analysis adjust for age and vaccination rates in the population on average, but they *do not* adjust for other factors such as comorbidities, access to healthcare, occupational exposures, etc. Failure to account for these additional characteristics may result in poorly-specified "target" IFR values. This limitation may make comparison of under-reporting rates between countries difficult; however, *relative* changes in under-reporting factors within and between countries may be more reliable, since many of these population characteristics related to IFR are time-invariant.

Figure B.3. Estimated under-reporting factors, adjusting for vaccination, during the first half of 2021

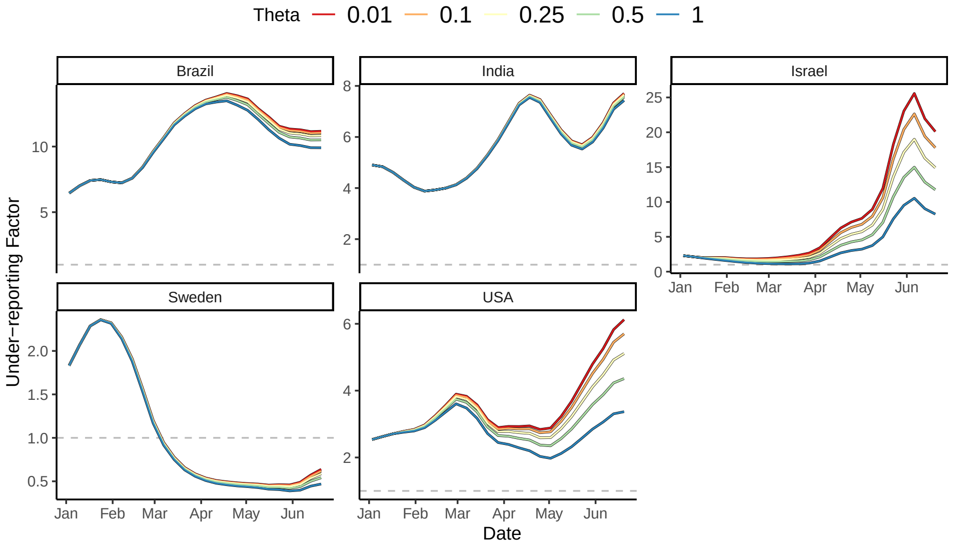

C Visualizations of resilience over time

This section provides additional visualizations of the resilience metrics over time each each of the countries.

Fig C.1 provides official COVID case counts, COVID-related deaths, and our infection fatality rate-adjusted estimates of the “true” number of weekly cases in each country per 100,000 residents. Corresponding estimated under-reporting factors are provided in **Fig C.2**. **Fig C.3** shows the number of COVID-related protests, battles, etc. recorded by ACLED. IHME-predicted hospitalization intakes and observed test and vaccine administration are presented in **Fig C.4**.

Figure C.1. Confirmed COVID cases and deaths and predicted true cases [1]

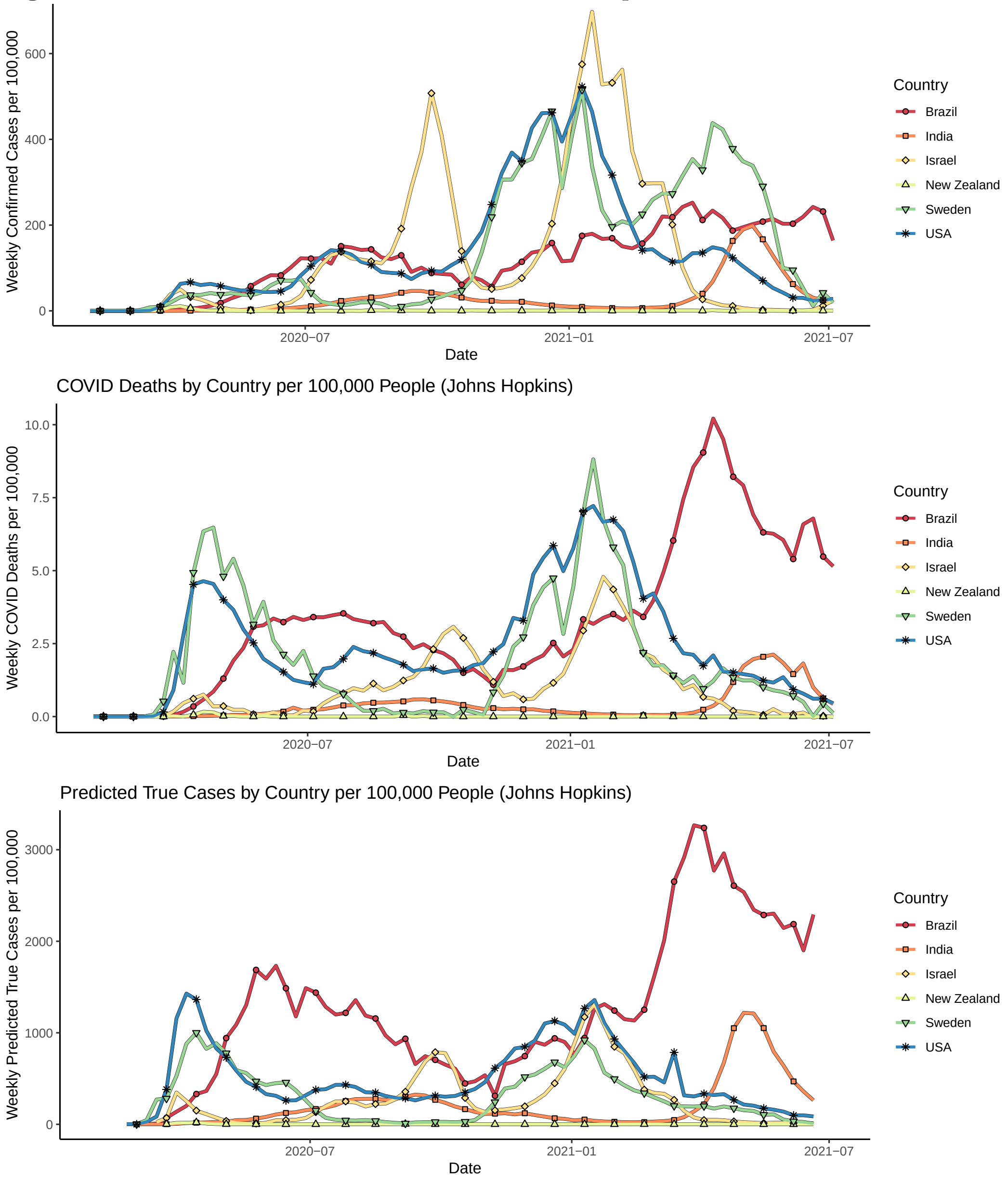

[1] Note that scales for the y-axes differ for each subfigure. Predicted true cases were calculated using the infection fatality rate adjustment method described in the main paper.

Figure C.2. Estimated case under-reporting factors over time [1]

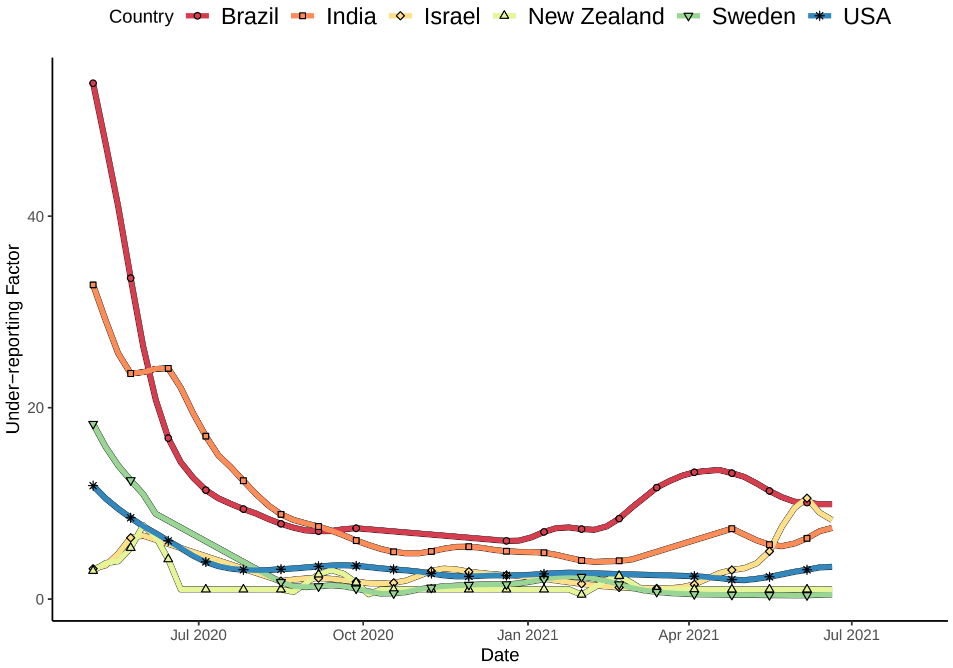

Figure C.3. Total number of COVID-related battles, explosions, protests, riots, strategic developments, or violence against civilians by country over time [1]

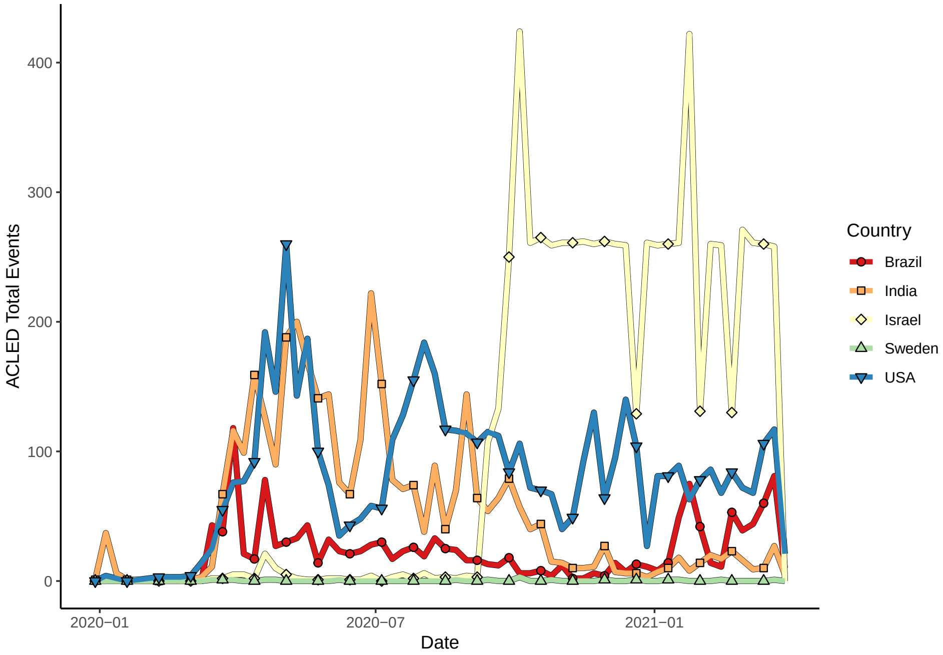

Figure C.4. Hospitalizations, vaccinations, and testing [1]

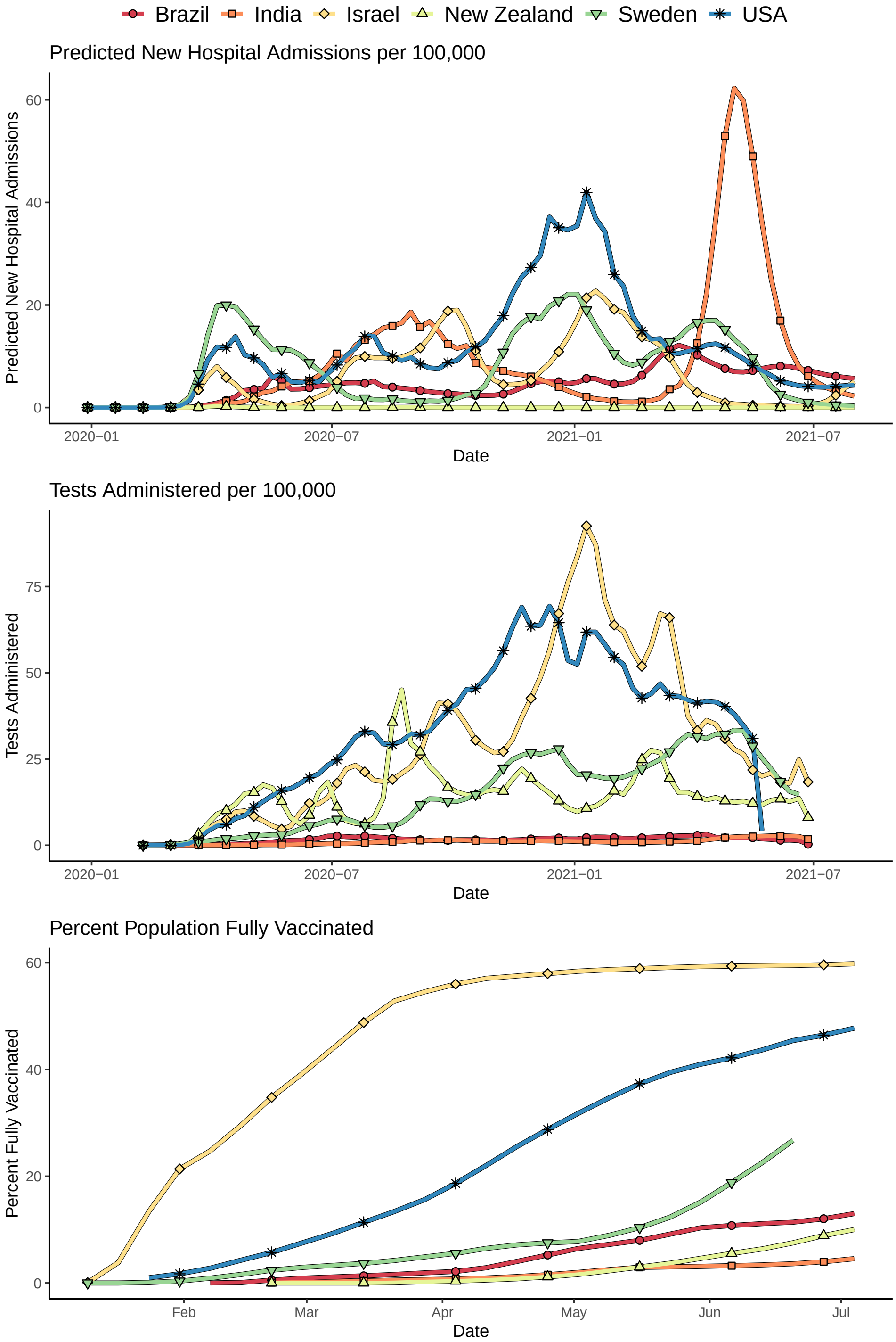

Figure C.5. Economic indicators over time by country [1]

(a) Gross domestic product (de-trended) and composite leading indicator

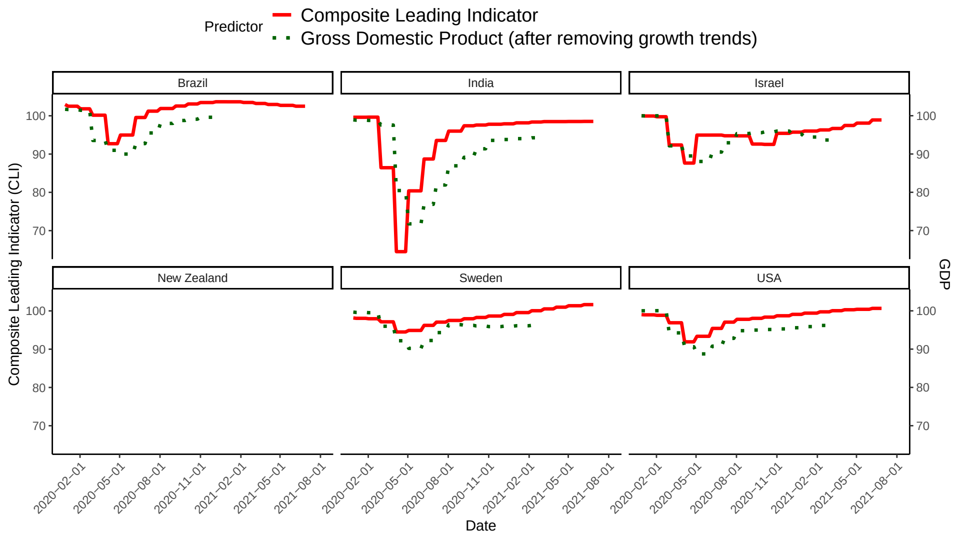

(b) Imports and Exports (millions of dollars)

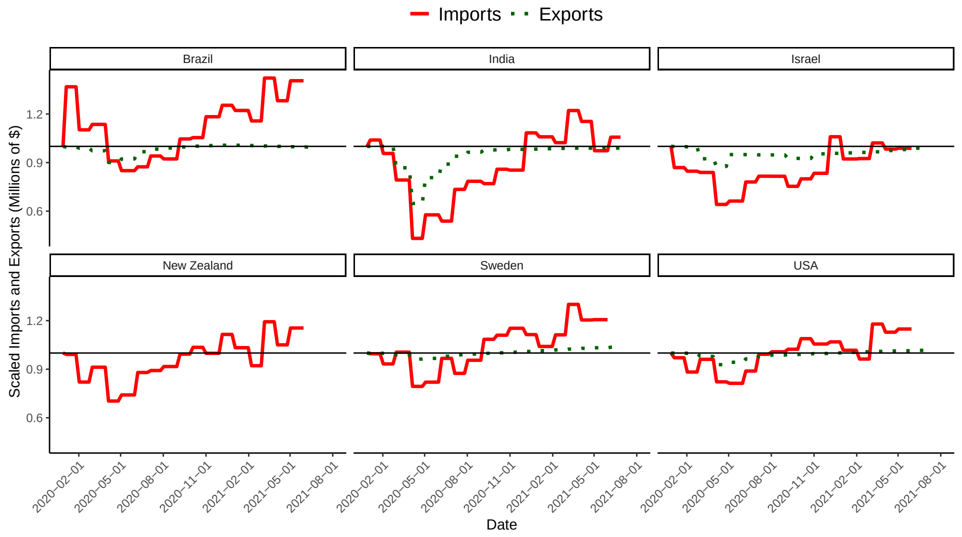

D Associations between resilience metrics

In this section, we provide additional metrics describing the relationships between resilience metrics over time. In **Fig D.1**, we provide the p-values related to pairwise bivariate Granger tests as described in the main paper. The goal of these statistical tests is to evaluate the relationship between a given predictor (columns) and time-lagged past values of other predictors (rows). For all countries, past corrected case counts are associated with future death rates. This is an artifact of the method used to estimate the corrected cases, which directly involves use of death weeks two weeks in the future. **Fig D.2** presents pairwise time series cross-correlations.

Figure D.1. P-values from bivariate Granger time series association tests[1]

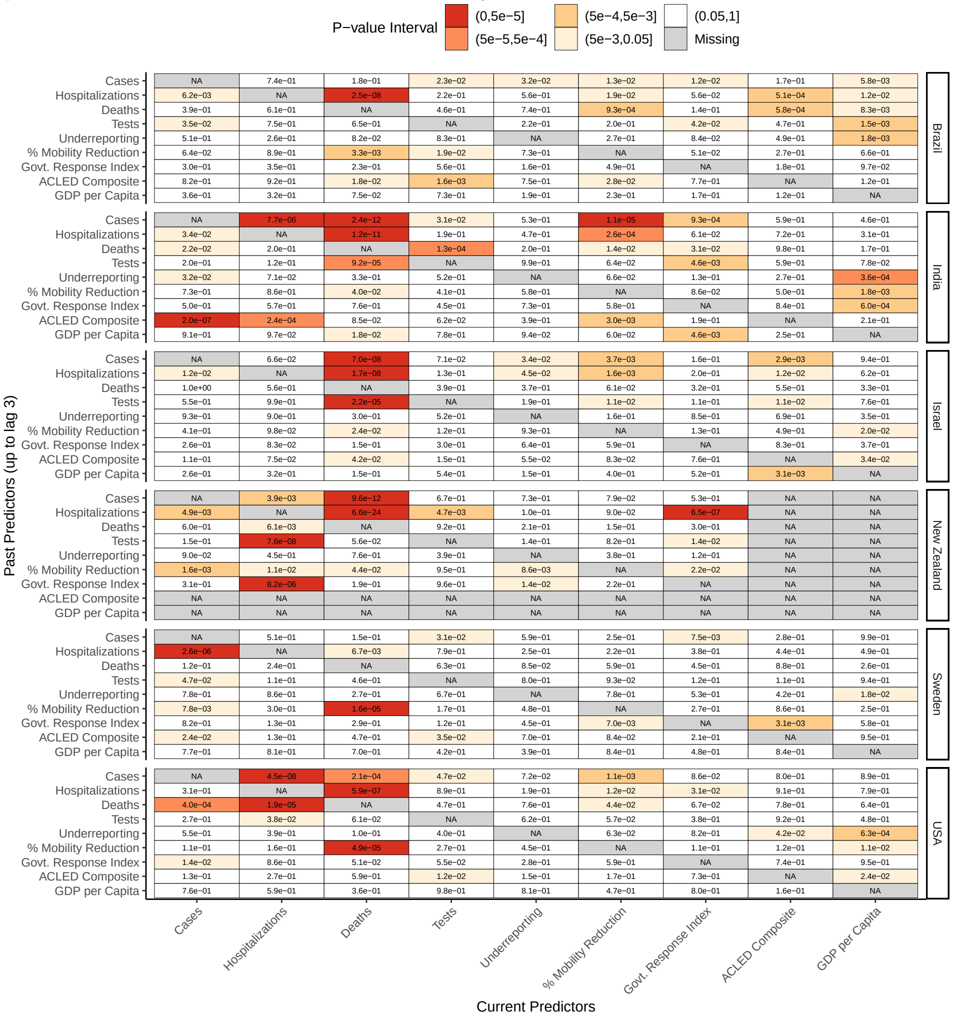

[1]Gray boxes correspond to missing data. Tests based on data for Quarter 2 in 2020 through Quarter 2 in 2021. For some resilience metrics (cases per 1000, deaths per 1000, hospitalizations per 1000, tests per 1000, ACLED events, under-reporting factors) , tests were applied after log transformation.

Figure D.2. Time series Spearman cross-correlations by country and quarter.[1]

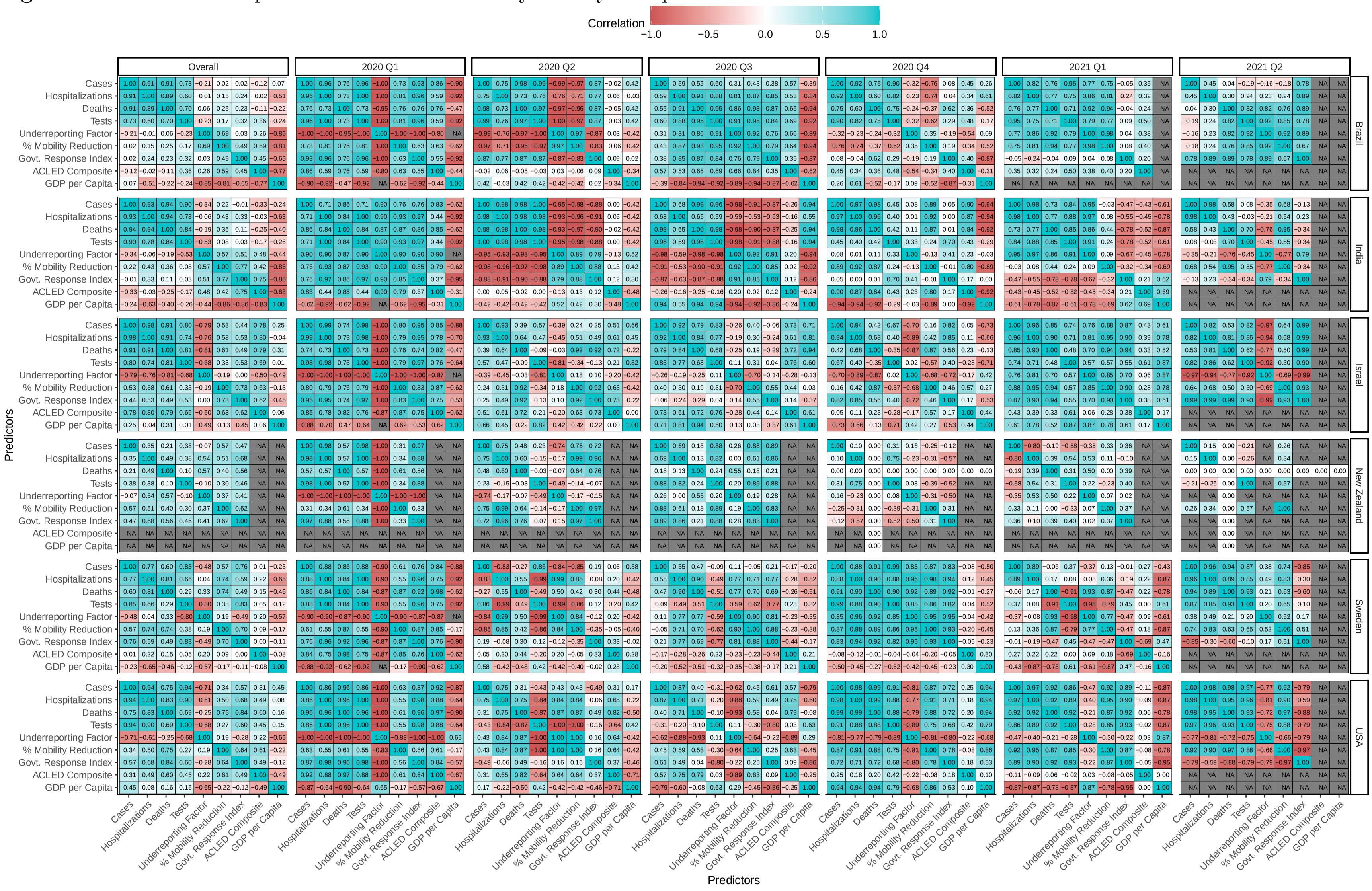

[1]Gray boxes correspond to missing data

E Additional modeling results

Table E.1. Spatio-temporal Bayesian mixed models for log-cases per 1000 with and without accounting for regional spatial correlations [1]

Characteristic	Model 1a	Model 1b	Model 2
	**Results for USA**		
Time-invariant			
log-Pop. density (100 per sq km)	-0.019 (-0.11, 0.08)	-0.012 (-0.10, 0.08)	0.003 (-0.05, 0.06)
Human development index (1 sd)	-0.014 (-0.08, 0.04)	-0.002 (-0.06, 0.05)	-0.006 (-0.04, 0.03)
Age over 60 (10 percent)	-0.086 (-0.29, 0.12)	-0.069 (-0.26, 0.13)	0.016 (-0.12, 0.15)
log-GDP per capita	0.003 (-0.03, 0.04)	0.010 (-0.02, 0.04)	0.006 (-0.01, 0.03)
Education (years)	-0.129 (-0.31, 0.05)	-0.155 (-0.32, 0.01)	-0.04 (-0.14, 0.06)
Time-varying			
Govt. policy index / 10, in previous week	**0.052** (0.04, 0.06)	-	-
log-Tests per 1000, in previous week	**0.091** (0.03, 0.15)	**0.105** (0.04, 0.17)	**0.096** (0.04, 0.15)
	**Results for Brazil**		
Time-invariant			
log-Pop. density (100 per sq km)	0.003 (-0.24, 0.24)	-0.004 (-0.22, 0.21)	-0.016 (-0.16, 0.14)
Human development index (1 sd)	0.167 (-0.02, 0.35)	0.143 (-0.02, 0.31)	0.092 (-0.02, 0.20)
Age over 60 (10 percent)	-0.283 (-0.61, 0.04)	-0.243 (-0.53, 0.05)	-0.166 (-0.44, 0.10)
log-GDP per capita	-0.023 (-0.09, 0.04)	-0.019 (-0.07, 0.04)	-0.016 (-0.05, 0.02)
Education (years)	-0.071 (-0.27, 0.12)	-0.058 (-0.23, 0.11)	-0.021 (-0.14, 0.09)
Time-varying			
Govt. policy index / 10, in previous week	-0.019 (-0.04, 0.00)	-	-
log-Tests per 1000, in previous week	**0.178** (0.07, 0.29)	**0.178** (0.07, 0.28)	**0.206** (0.12, 0.30)
	**Results for India**		
Time-invariant			
log-Pop. density (100 per sq km)	-0.035 (-0.17, 0.10)	-0.019 (-0.13, 0.09)	-0.001 (-0.03, 0.03)
Human development index (1 sd)	0.085 (-0.09, 0.26)	0.060 (-0.08, 0.20)	0.032 (0.00, 0.07)
Age over 60 (10 percent)	**0.781** (0.20, 1.37)	**0.547** (0.09, 1.01)	0.069 (-0.10, 0.23)
log-GDP per capita	-0.016 (-0.07, 0.04)	-0.017 (-0.06, 0.02)	0.006 (-0.02, 0.03)
Education (years)	-0.011 (-0.10, 0.08)	0.001 (-0.07, 0.07)	-
Time-varying			
Govt. policy index / 10, in previous week	**0.091** (0.06, 0.13)	-	-
log-Tests per 1000, in previous week	**0.158** (0.08, 0.24)	**0.140** (0.08, 0.20)	**0.073** (0.05, 0.10)
	**Results for Sweden**		
Time-invariant			
log-Pop. density (100 per sq km)	-0.159 (-0.76, 0.44)	0.013 (-0.41, 0.43)	0.079 (-0.25, 0.43)
Human development index (1 sd)	0.004 (-0.10, 0.11)	-0.015 (-0.09, 0.06)	-0.012 (-0.05, 0.02)
Age over 60 (10 percent)	-0.019 (-0.34, 0.30)	-0.104 (-0.33, 0.12)	-0.062 (-0.21, 0.09)
log-GDP per capita	-0.054 (-0.17, 0.06)	-0.012 (-0.09, 0.07)	0.010 (-0.04, 0.06)
Education (years)	-	-	
Time-varying			
Govt. policy index / 10, in previous week	**-0.197** (-0.33, -0.06)	-	-
log-Tests per 1000, in previous week	**-0.168** (-0.26, -0.08)	-0.039 (-0.11, 0.03)	-0.008 (-0.07, 0.05)

[1] This table presents parameter estimates and 95% credible intervals from several varieties of mixed models for log-cases per 1000 for each of four countries separately. Model 1 contains a random intercept for region (administrative level 1) and an autoregressive error structure over time within each region. The column "Model 1a" provides results from fitting Model 1 to data where missing case counts (nearly always occurring prior to the first reported case for each region) are excluded. "Model 1b" provides results from fitting Model 1 to data where the missing cases count values are replaced with zeros and where government policy is excluded as a predictor from the model. "Model 2" uses the same data as "Model 1b" but also incorporates the spatial correlation between neighboring states within each country. Both Models 1 and 2 also include an 8-degree natural spline of calendar time. Population density (for Sweden) and governmental policy (all countries) were excluded from Model 2 due to missing data in these regions making the spatial correlation not able to be computed.

F Economic analysis

F.1 Input-Output model

In this section, we give a brief description of an Input-Output (IO) model. An IO model is created starting from observations for a particular area, usually a nation or a region. The economic activities of an area are classified into sectors/industries. We use the International Standard Industrial Classification (ISIC) revision 3 industry classification in this work, a standard defined by UN Statistical Division [15]. National data using different classifications are mapped into ISIC code. See **Table F.1** for the ISIC sectors' definitions and **Table F.2** for the ISIC sections, an aggregation of sectors.

The Input-Output relationships can be summarized as follows. Suppose the quantity x_i is the total production of sector i and f_i the total final demand of sector's i products. In that case, we can represent the relationship between industries, i.e., how much one industry is selling to other sectors, by the following equation:

$$x_i = z_{i,1} + \cdots + z_{i,j} + \cdots + z_{i,n} + f_i = \sum_{j=1}^{n} z_{i,j} + f_i$$

where $z_{i,j}$ is the sale of sector i to sector j. We can express the relations between industries in matrix notation:

$$\boldsymbol{x} = \boldsymbol{Z}\boldsymbol{i} + \boldsymbol{f},$$

The total output of sector j is denoted x_j. The ratio between z_{ij} and x_j is called the *technical coefficient*. The technical coefficient represents the amount of input from sector i expressed in dollars required to produce a one-dollar output in the industry j. We can rewrite F.1 as:

$$x_i = a_{i,1}x_i + \cdots + a_{i,j}x_i + \cdots + a_{i,n}x_i + f_i.$$

If we group the x on the left side we have:

$$\begin{aligned}
(1 - a_{11})x_1 \quad & - \cdots - a_{1,i}x_i - \cdots - a_{i,n}x_n = f_1 \\
\vdots \quad & \\
-a_{i1}x_1 \quad & - \cdots + (1 - a_{1,i})x_i - \cdots - a_{i,n}x_n = f_i \\
\vdots \quad & \\
-a_{i1}x_1 \quad & - \cdots - a_{1,i}x_i - \cdots + (1 - a_{i,n})x_n = f_n
\end{aligned}$$

that in matrix notation corresponds to:

$$(\boldsymbol{I} - \boldsymbol{A})\boldsymbol{x} = \boldsymbol{f},$$

For a given $\boldsymbol{f}$, we solve for $\boldsymbol{x}$:

$$\boldsymbol{x} = (\boldsymbol{I} - \boldsymbol{A})^{-1}\boldsymbol{f} = \boldsymbol{L}\boldsymbol{f},$$

to obtain $\boldsymbol{L}$, known as the *Leontief inverse*. Each element of $\boldsymbol{L}$, l_{ij}, corresponds to $\partial x_i/\partial f_j$. The Leontief Inverse allows the computation of an economic shock's direct and indirect effects on an economy.

F.2 Industry specific infection weight

Workers employed in distinct industrial sectors have different probabilities of contracting COVID-19. For example, employees in the health care industry have a

higher probability of contracting the virus than workers in the IT sector that have the option of teleworking. Industry infection weights are computed to quantify the effect of the industry-specific working environments on the workers' probability of contracting COVID-19. The industry infection weights are then applied to the national time series of COVID cases to determine the industry-specific COVID cases time series.

We use two data sources to compute the weights. The first one is published by the California Department of Public Health [16]: following California rules, employers are required to report workplace COVID-19 outbreaks to their local health department. The data are published using the Census Industry Code.

The second data set is from the Employment Development Department of The State of California, and it contains monthly employment data coded using the NAICS industry classification. Using the "Census Industry Code List" published by Census [17] we mapped the California outbreaks data to the NAICS industry classification. The two datasets, outbreaks and employment, are finally mapped from NAICS to the ISIC industry classification standard, the same used in the IO data [18]. From the reclassified industry data we compute c_i, the fraction of infected workers in industry i. The industry infection weight r_i is defined as:

$$r_i = \frac{c_i - \sum_{i \in I} c_i}{\sum_{i \in I} c_i} + 1. \qquad (Eq.\ S4)$$

We assume that the estimated industry infection weight r_i is the same across nations.

F.3 Workforce reduction impact analysis

The first step to analyzing the economic impact of workforce reduction due to the pandemic is to generate the distribution of workers for each industrial sector for each country. We are using the employment distribution data derived from the world IO model. The underlying data are collected from national and European statistical bureaus [**?**, see]appendix B]WOI4. The distribution of workers across the industrial sector is quite different across the countries we analyzed. **Fig F.2** depicts the distribution of workers for the top 30 industrial sectors of each country part of the impact analysis. The outer ring shows the distribution of workers across sectors while the inner ring aggregates the fractions by economic segment. Economic sectors and sections are listed in **Tables F.1 and F.2**.

The second step is to compute the sensitivity of each economic sector from the workforce. The production requirements of each industry include, for example, commodity, labor, capital, and energy inputs. The Socio Economic Account (SEA) data table of World Input-Output Database (WIOD) limits us to two production factors for each sector i: labor L_i and capital K_i expressed in millions of the national currency. The sensitivity s_i of the sector i to the workforce is the ratio between labor and the total production requirements: $s_i = \frac{L_i}{L_i + K_i}$.

Using the national daily time-series data of the number of newly infected people, we determine the number of infected people per industry sector i at time t, $\Psi_i(t)$ as:

$$\Psi_i(t) = \delta \ \mathrm{inf}(t) \ \frac{r_i w_i}{\sum_{i \in I} r_i w_i} \qquad (Eq.\ S5)$$

where δ is the fraction of working population over the total population, w_i is the number of workers in the sector i, $\mathrm{inf}(t)$ is the number of new people infected at time t, and r_i is the industry specific infection weight defined in *Eq. S4*.

Assuming an average illness period of 3 weeks, the change in production for industry i at time t, $cp_i(t)$, is determined by the change of workforce due the illness multiplied by

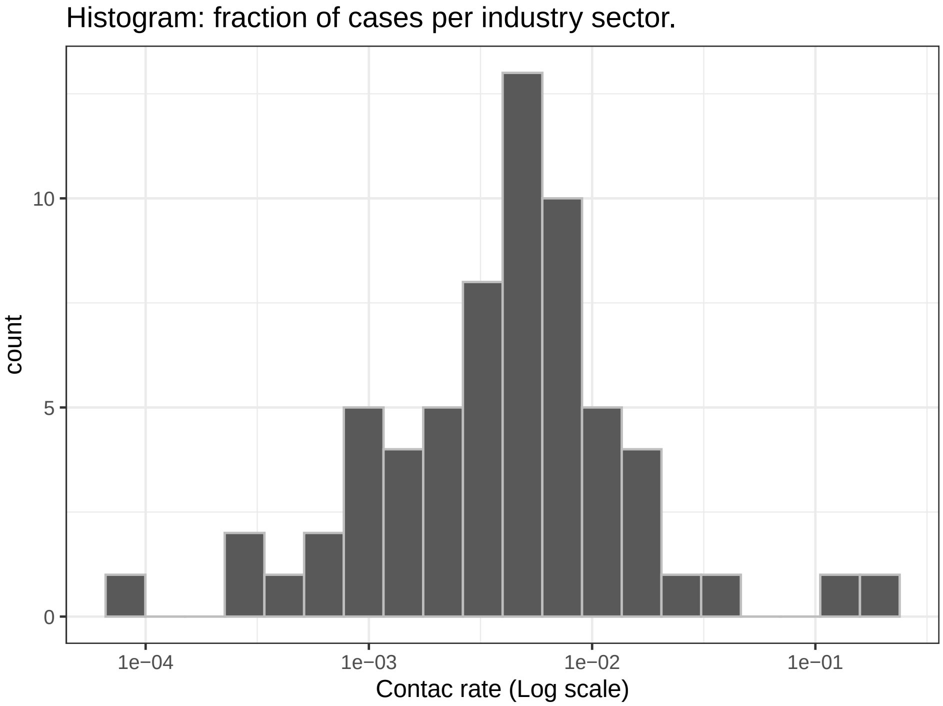

Figure F.1. Histogram of the fraction of cases per industry sector. The California Department of Public Health collected the number of cases per industry in 2021. The California Development Department provides the number of employees per industry. From the 2021 monthly observations, we computed the annual average. The fraction of cases per industry is in the Log scale. Cases and employees are mapped to ISIC-2017 industry sectors.

s_i, the sensitivity to the output of sector i to the labour factor:

$$cp_i(t) = \sum_{m=0}^{20} \Psi_i(t-m)\ s_i \frac{p_i}{365}, \qquad (\textit{Eq. S6})$$

where p_i is the production level of sector i. We assume that δ, p_i, and s_i are constant during the analysis period.

Finally, we compute the economic drop in production $\boldsymbol{d}(t)$ for each sector, by propagating the shock across the economy of interconnected sectors through the input-output multipliers:

$$\boldsymbol{d}(t) = \boldsymbol{L}\ \boldsymbol{cp}(t),$$

where $\boldsymbol{L}$ is the Leontief Inverse matrix, $\boldsymbol{cp}(t) = (cp_1(t), ..., cp_n(t))$ is the vector of the change in production as in *Eq. S6*. The drop in production, $\boldsymbol{d}(t)$, incorporates both the direct and indirect effects of the shock on the economy.

Fig F.4 depicts the economic impact due to pandemic-induced workforce reduction. The distribution of losses across the industrial sectors is computed following the above methodology.

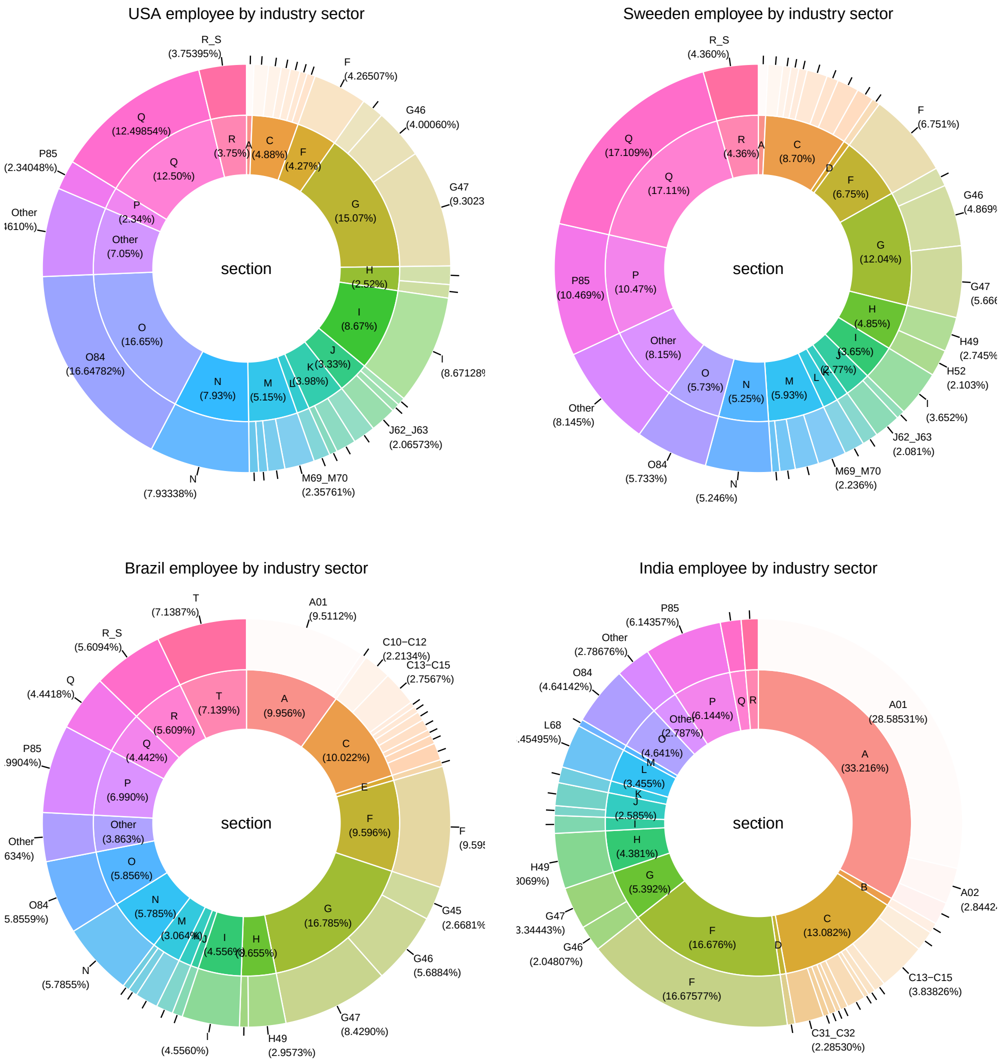

Figure F.2. Employment by sector. Refer to **Tables F.1 and F.2** for the sector names. Sections are groups of sectors, for example "A" stands for Agriculture. For example, the fraction of employee in the Agriculture sectors in US and Sweden is quite small if compared to India and Brazil.

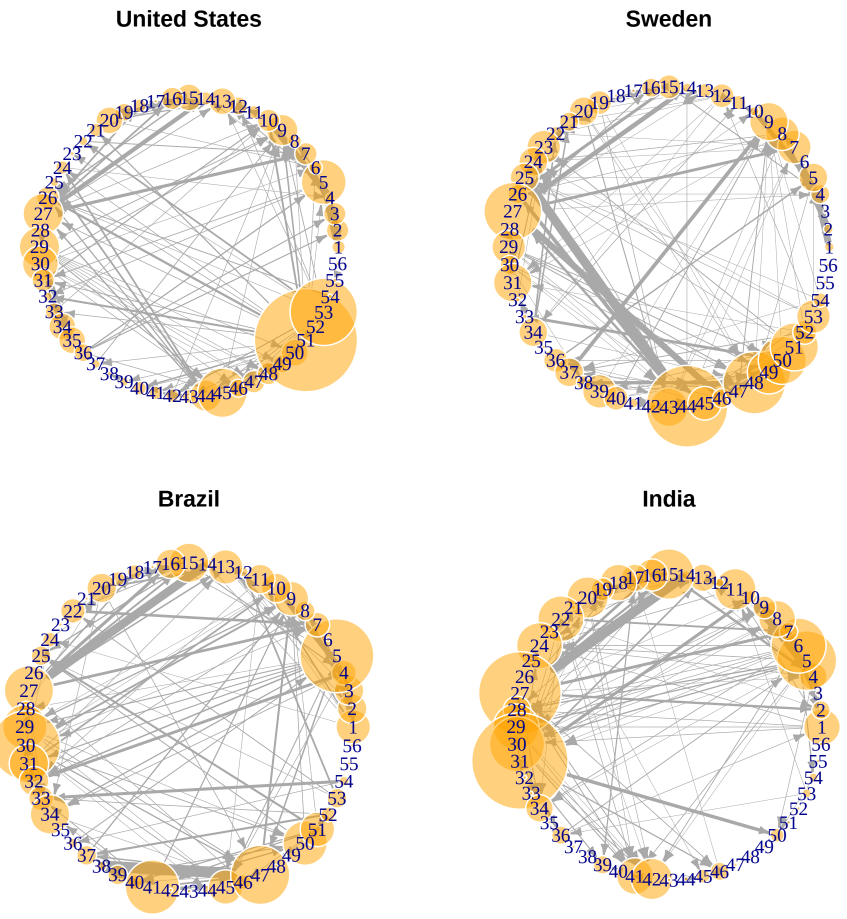

Figure F.3. Input-output tables network representation. The input-output table is pictured as a directed weighted graph. Each vertex represents an economic sector, and it is labeled with an index: see Table F.1 for a complete description of the sectors. The size of each vertex is proportional to the degree of the vertex, i.e., the number of its adjacent edges. The thickness of the graph edges is proportional to the connection strengths between sectors. The graph shows the most important connections between sectors: only the top 5 percent of the edges' weight distribution is visualized in the network.

Ind	Id	Description
1	A01	Crop and animal production, hunting and related service activities
2	A02	Forestry and logging
3	A03	Fishing and aquaculture
4	B	Mining and quarrying
5	C10.C12	Manufacture of food products, beverages and tobacco products
6	C13.C15	Manufacture of textiles, wearing apparel and leather products
7	C16	Manufacture of wood and of products of wood and cork, except furniture; manufacture of articles of straw and plaiting materials
8	C17	Manufacture of paper and paper products
9	C18	Printing and reproduction of recorded media
10	C19	Manufacture of coke and refined petroleum products
11	C20	Manufacture of chemicals and chemical products
12	C21	Manufacture of basic pharmaceutical products and pharmaceutical preparations
13	C22	Manufacture of rubber and plastic products
14	C23	Manufacture of other non-metallic mineral products
15	C24	Manufacture of basic metals
16	C25	Manufacture of fabricated metal products, except machinery and equipment
17	C26	Manufacture of computer, electronic and optical products
18	C27	Manufacture of electrical equipment
19	C28	Manufacture of machinery and equipment n.e.c.
20	C29	Manufacture of motor vehicles, trailers and semi-trailers
21	C30	Manufacture of other transport equipment
22	C31_C32	Manufacture of furniture; other manufacturing
23	C33	Repair and installation of machinery and equipment
24	D35	Electricity, gas, steam and air conditioning supply
25	E36	Water collection, treatment and supply
26	E37.E39	Sewerage; waste collection, treatment and disposal activities; materials recovery; remediation activities and other waste management services
27	F	Construction
28	G45	Wholesale and retail trade and repair of motor vehicles and motorcycles
29	G46	Wholesale trade, except of motor vehicles and motorcycles
30	G47	Retail trade, except of motor vehicles and motorcycles
31	H49	Land transport and transport via pipelines
32	H50	Water transport
33	H51	Air transport
34	H52	Warehousing and support activities for transportation
35	H53	Postal and courier activities
36	I	Accommodation and food service activities
37	J58	Publishing activities
38	J59_J60	Motion picture, video and television programme production, sound recording and music publishing activities; programming and broadcasting activities
39	J61	Telecommunications
40	J62_J63	Computer programming, consultancy and related activities; information service activities
41	K64	Financial service activities, except insurance and pension funding
42	K65	Insurance, reinsurance and pension funding, except compulsory social security
43	K66	Activities auxiliary to financial services and insurance activities
44	L68	Real estate activities
45	M69_M70	Legal and accounting activities; activities of head offices; management consultancy activities
46	M71	Architectural and engineering activities; technical testing and analysis
47	M72	Scientific research and development
48	M73	Advertising and market research
49	M74_M75	Other professional, scientific and technical activities; veterinary activities
50	N	Administrative and support service activities
51	O84	Public administration and defence; compulsory social security
52	P85	Education
53	Q	Human health and social work activities
54	R_S	Other service activities
55	T	Activities of households as employers; undifferentiated goods- and services-producing activities of households for own use
56	U	Activities of extraterritorial organizations and bodies

Table F.1. ISIC economic sectors reference table. In the first column, the economic sector index (used in **Fig F.3**), in the second column the economic sector identifier, and in the third column the sector definition.

Section	Division	Description
A	01–03	Agriculture, forestry and fishing
B	05–09	Mining and quarryingv
C	10–33	Manufacturing
D	35	Electricity, gas, steam and air conditioning supply
E	36–39	Water supply; sewerage, waste management and remediation activities
F	41–43	Construction
G	45–47	Wholesale and retail trade; repair of motor vehicles and motorcycles
H	49–53	Transportation and storage
I	55–56	Accommodation and food service activities
J	58–63	Information and communication
K	64–66	Financial and insurance activities
L	68	Real estate activities
M	69–75	Professional, scientific and technical activities
N	77–82	Administrative and support service activities
O	84	Public administration and defence; compulsory social security
P	85	Education
Q	86–88	Human health and social work activities
R	90–93	Arts, entertainment and recreation
S	94–96	Other service activities
T	97–98	Activities of households as employers; undifferentiated goods and services-producing activities of households for own use
U	99	Activities of extraterritorial organizations and bodies

Table F.2. ISIC sections: The individual categories of ISIC are aggregated into 21 general sections.

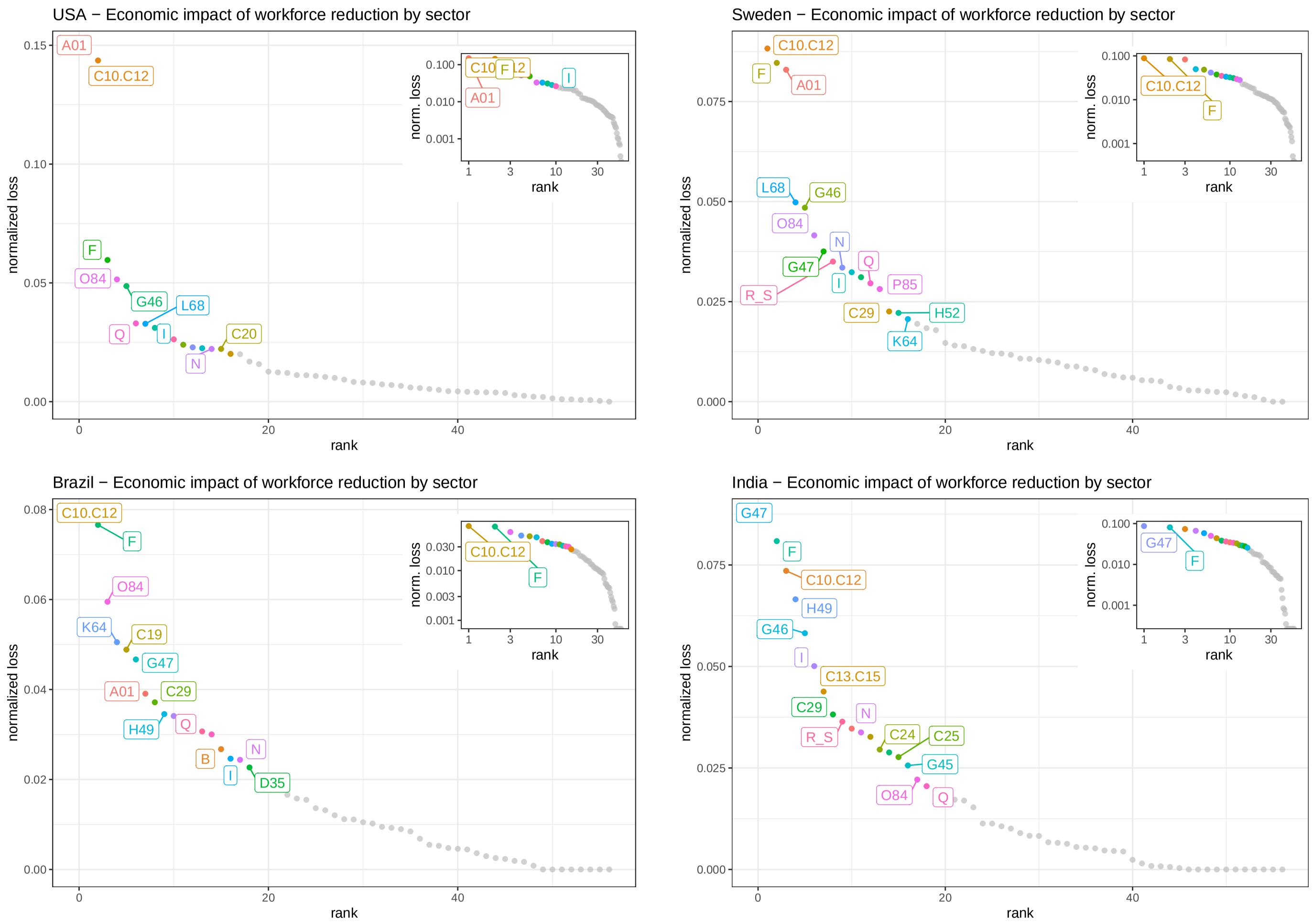

Figure F.4. Economic impact of workforce reduction due to the pandemic in USA, Sweden, Brazil and India. Each plot shows the rank distribution of the losses by economic sector. Losses are normalized to 1, refer to **Table F.1** for the label interpretation.

References

1. Badr HS, Zaitchik BF, Kerr GH, Nguyen NL, Chen YT, Hinson P, et al. Unified real-time environmental-epidemiological data for multiscale modeling of the COVID-19 pandemic. medRxiv. 2021;doi:10.1101/2021.05.05.21256712.

2. Clionadh R, Andrew L, Håvard H, Joakim K. Introducing ACLED-Armed Conflict Location and Event Data. Journal of Peace Research. 2010;47(5):651–660.

3. Ritchie H, Mathieu E, Rodés-Guirao L, Appel C, Giattino C, Ortiz-Ospina E, et al. Coronavirus Pandemic (COVID-19). Our World in Data. 2020;.

4. Thomas H, Noam A, Rafael G, Beatriz K, Anna P, Toby P, et al. A global panel database of pandemic policies (Oxford COVID-19 Government Response Tracker). Nature Human Behaviour. 2021;5:529–538.

5. IHME. Data and Forecast Repository; 2021. Available from: `http://www.healthdata.org/covid/data-downloads`.

6. OECD. Composite leading indicator (CLI) (indicator). 2021;doi:10.1787/4a174487-en.

7. WTO. World Trade Organization. International trade statistics; 2021. Available from: `https://timeseries.wto.org`.

8. Global Data Lab. Subnational SDG Dashboard; 2021. Available from: `https://globaldatalab.org/shdi/msch/`.

9. Kummu M, Taka M, Guillaume JHA. Gridded global datasets for Gross Domestic Product and Human Development Index over 1990-2015. Scientific Data. 2018;5:1–15. doi:10.1038/sdata.2018.4.

10. CIESIN - Columbia University. Gridded Population of the World, Version 4 (GPWv4): Administrative Unit Center Points with Population Estimates; 2016. Available from: `http://dx.doi.org/10.7927/H4F47M2C`.

11. BLS. U.S. Bureau of Labor Statistics; 2021.

12. Timmer MP, Dietzenbacher E, Los B, Stehrer R, de Vries GJ. An Illustrated User Guide to the World Input–Output Database: the Case of Global Automotive Production. Review of International Economics. 2015;23:575–605.

13. Lau H, Khosrawipour T, Kocbach P, Ichii H, Bania J, Khosrawipour V. Evaluating the massive underreporting and undertesting of COVID-19 cases in multiple global epicenters. Pulmonology. 2020;27(2):110–115. doi:10.1016/j.pulmoe.2020.05.015.

14. O'Driscoll M, Ribeiro Dos Santos G, Wang L, Cummings DAT, Azman AS, Paireau J, et al. Age-specific mortality and immunity patterns of SARS-CoV-2. Nature. 2021;590(7844):140–145. doi:10.1038/s41586-020-2918-0.

15. Nations NYU. International Standard Industrial Classification of All Economic Activities Revision 3, Series M: Miscellaneous Statistical Papers, No. 4 Rev. 3;.

16. Health C, Services H. Total COVID-19 Outbreaks by Industry; 2022. Available from: `https://www.cdph.ca.gov/Programs/CID/DCDC/Pages/Immunization/ncov2019.aspx`.

17. Bureau USC. Census 2017 Industry Code List with Crosswalk; 2021. Available from: https://www2.census.gov/programs-surveys/demo/guidance/industry-occupation/2017-industry-code-list-with-crosswalk.xlsx.

18. Liao S, Kim IS, Miyano S, Zhu F. concordance: Product Concordance; 2020. Available from: https://CRAN.R-project.org/package=concordance.
